# Method for Observing SMOKing and vaping bEhaviours (MOSMOKE): development and validation of a systematic observation tool

**DOI:** 10.1136/bmjopen-2025-105510

**Published:** 2025-07-13

**Authors:** Jack S Benton, Sofia Berg, Arbaz Kapadi, Neil Bendel, Julie Jerram, David P French

**Affiliations:** 1 Department of Geography, School of Environment, Education and Development, The University of Manchester, Manchester, UK; 2 Manchester Centre for Health Psychology, Division of Psychology & Mental Health, School of Health Sciences, The University of Manchester, Manchester, UK; 3 Department of Public Health, Manchester City Council, Manchester, UK

**Keywords:** Smoking Reduction, PUBLIC HEALTH, Psychometrics

## Abstract

**Abstract:**

**Objective:**

Policies and interventions increasingly aim to reduce smoking in outdoor public spaces, but evidence on factors influencing smoking in specific locations remains limited. Systematic observation can unobtrusively assess behaviours in environmental contexts, reducing biases from self-report. This study aims to develop and test the reliability and validity of MOSMOKE (Method for Observing SMOKing and vaping bEhaviours): a new tool for assessing the number of people holding or inhaling a cigarette or vape in public spaces.

**Design:**

MOSMOKE was adapted from a previously validated observation tool for assessing physical activity and well-being behaviours. Following piloting and refinement, inter-rater reliability for assessing smoking, vaping and age group classification was analysed using intraclass correlation coefficients (ICCs). A main study assessed criterion-related validity through 32 hours of observations over 4 days. A 2×2 study design was used, with four sites selected that varied by two environmental characteristics: presence of a smoking bin and adjacency to an office block.

**Setting:**

Four public spaces in Manchester, UK.

**Results:**

Inter-rater reliability was ‘good’ (ICCs>0.75; n=4) or ‘excellent’ (ICCs>0.90; n=2) for smoking behaviours, and mostly ‘good’ (n=4) or ‘excellent’ (n=1) for vaping behaviours. Observed differences in smoking and vaping behaviours across sites aligned with prespecified hypotheses that smoking would be more prevalent near smoking bins (p=0.02) and office entrances (p=0.006), supporting criterion-related validity.

**Conclusions:**

This study provides preliminary evidence that MOSMOKE is a reliable and valid tool for unobtrusively assessing smoking and vaping in public spaces. It can be used to evaluate policies and interventions targeting smoking or vaping in specific environmental contexts. MOSMOKE is freely available, with a detailed manual to support its use.

**STRENGTHS AND LIMITATIONS OF THIS STUDY**

- This is the first study to develop and test an observation tool (Method for Observing SMOKing and vaping bEhaviours) specifically designed to assess smoking and vaping behaviours in public spaces.
- The tool demonstrated high inter-rater reliability with minimal observer training, suggesting it can be applied consistently in real-world settings.
- The study did not include participant surveys, so some behaviours may have been misclassified (eg, heated tobacco products vs vaping).
- Reliability estimates for coding age groups were lower than expected, partly due to limited contextual cues and less emphasis on training for this variable.
- Further research is needed to test the tool’s applicability across diverse urban environments and in natural experimental studies to evaluate its sensitivity to change.

## Introduction

 Tobacco smoking remains the leading cause of preventable death and disease worldwide, contributing to over 8 million deaths annually.^1^ To combat this, a range of public health strategies have been implemented to reduce smoking prevalence and protect non-smokers from secondhand smoke exposure. A key framework guiding these efforts is the WHO’s MPOWER policy package,^2^ which recommends measures such as increasing tobacco taxes, creating smoke-free environments, and bans on tobacco advertising, promotion and sponsorship.

Building on these global tobacco control strategies, many countries worldwide are increasingly implementing policies and interventions to reduce smoking in outdoor public spaces, including parks, urban squares, transport hubs and beaches.^3^ Examples include outdoor smoking bans (eg, New York City’s smoke-free parks and beaches law^4^), public awareness campaigns and advertisements (eg, Qatar’s national antitobacco health awareness campaigns including advertisements in outdoor locations^5^), signage (eg, a UK local authority displaying children’s signs asking people not to ‘smoke where we play’ in parks^6^) and modifications to the physical environment (eg, Shanghai’s outdoor smoking zones aimed at discouraging people from smoking while walking^7^).

However, robust evidence on the effectiveness of outdoor smoking interventions remains limited. Surveys are commonly used to assess smoking behaviours^8^, but they are often limited by self-report biases, low response rates and difficulties capturing smoking behaviours in specific environmental contexts.^9^ Some studies have evaluated the effectiveness of indoor smoking interventions using objective measures of secondhand smoke exposure (eg, airborne nicotine^10^), and these methods are now being applied outdoors (eg, outdoor areas of cafés and restaurants^11^). However, these environmental measures provide limited insight into actual changes in smoking behaviour. As a result, it remains unclear whether these place-based interventions are effective at influencing smoking behaviours in outdoor settings.

Systematic observation (ie, direct observations of behaviour using predetermined criteria) is a promising method for assessing how smoking behaviours vary across different environmental contexts.^9^ It can, therefore, offer useful insights into how environments influence smoking behaviours. Observations can be unobtrusive, where participants are not aware they are being assessed, and thus can be carried out without participant burden and selection or reactivity biases typically associated with self-report.

Our scoping review of the literature identified 13 studies that have used observational methods to assess smoking in public spaces.^412^,^23^ However, these studies varied considerably in the specific smoking behaviours assessed and the observational scanning methods used. Additionally, only two studies reported formal assessment of inter-rater reliability (agreement between independent observers).^4 23^ This lack of standardisation limits the comparability and robustness of findings across studies and locations.

Further, only one study attempted to observe e-cigarette use (commonly referred to as ‘vaping’).^14^ Vaping has become widespread internationally, in part driven by innovations in vape pen design and nicotine flavouring. Concerns about the health effects of inhaling e-cigarette aerosol and exposure to secondhand aerosol are growing.^24^ The WHO has recommended that e-cigarettes be regulated similarly to tobacco products,^25^ and many governments are introducing policies such as marketing restrictions, flavour bans and age verification. Therefore, systematically monitoring vaping behaviours in public spaces is crucial to evaluate these emerging policies amid a rapidly evolving regulatory landscape.

To address these gaps, we report here on the development and testing of Method for Observing SMOKing and vaping bEhaviours (MOSMOKE)—a new systematic observation tool for counting the number of people holding or inhaling a cigarette or vape in public spaces. MOSMOKE also includes an environmental audit component for assessing cigarette and vape litter (eg, cigarette butts, vape cartridges), which are non-biodegradable and environmentally toxic.

### Aims and objectives

This study aimed to develop the MOSMOKE tool and test its reliability and validity. The specific objectives were to: (1) develop a new systematic observation tool for assessing smoking and vaping behaviours in public spaces; (2) assess inter-rater reliability between pairs of observers and (3) evaluate criterion-related validity by examining differences in smoking and vaping behaviours in relation to environmental characteristics hypothesised to be associated with these behaviours.

## Methods

### Setting

The study was conducted in St Peter’s Square, a busy public square in central Manchester, UK. Manchester is an ideal setting to test this new methodology because of its above-average adult smoking prevalence rate; the latest published UK Official for National Statistics data for 2023 suggest that 12.5% of adults aged 18 years and over in Greater Manchester currently smoke cigarettes, compared with the 11.9% national average in the UK.^26^ St Peter’s Square was chosen as it provided sufficient footfall to collect a large volume of data. Further, Manchester City Council has identified this square as a potential site for future outdoor smoke-free public spaces.^27^

### Development and piloting of MOSMOKE

To address the lack of standardised and psychometrically tested tools for systematically observing smoking and vaping behaviours in public spaces, we based the development of MOSMOKE on the validated Method for Observing pHysical Activity and Well-being (MOHAWk).^26^ MOHAWk assesses physical activity levels (sedentary, walking and vigorous), two other wellbeing-related behaviours (social interactions and taking notice of the environment) and demographic characteristics (gender, age group and ethnic group) in public spaces. It has demonstrated high inter-rater reliability and criterion-related validity^26^ and has been successfully used in four published natural experimental studies of environmental interventions.^28^,^31^ MOHAWk was chosen both for its strong psychometric properties and our extensive prior experience using it during over 1000 hours of observation in similar urban environments.

To adapt MOHAWk for the purpose of assessing smoking and vaping, four new behavioural codes were added to code individuals either holding a cigarette or vape, or actively inhaling a cigarette or vape (indicated by arm-to-mouth movement). This preliminary version of MOSMOKE was piloted by two observers (JSB and SB) over 3 days in St Peter’s Square. Pilot testing showed that attempting to code all original MOHAWk behaviours and demographic variables alongside the new smoking and vaping behaviours was not feasible and led to unreliable data. Therefore, only the age group was retained as a demographic variable, given its importance for identifying age-related patterns in smoking and vaping.^32^

### MOSMOKE procedures

The MOSMOKE tool was used to systematically code smoking and vaping behaviours of all individuals entering a clearly defined target area during pre-specified observation periods. Before data collection began, all observers visited the site together to agree on the exact boundaries of the target area, ensuring consistency in determining which individuals fell within the designated space. This process was crucial to avoid ambiguity when coding behaviours, as only individuals and behaviours within the target area were included in the observations.

During each observation period, observers used MOSMOKE to code the following:

1. Smoking and vaping behaviours:

- Holding a cigarette.
- Holding a vape.
- Inhaling a cigarette (identified by arm-to-mouth movement).
- Inhaling from a vape (identified by arm-to-mouth movement).

1. Estimated age group (based on visual cues such as appearance, clothing and mobility):

- Infant (baby or toddler in a pram, sling or other carrier).
- Child (appears to be up to 12 years old).
- Teen (appears to be 13–19 years old).
- Adult (appears to be 20–74 years old).
- Older Adult (appears to be 75+ years old).

Large groups (more than 10 individuals) were excluded from observations. This was to minimise the risk of missing smoking or vaping behaviours, as it was difficult to accurately observe and code behaviours within such groups while simultaneously monitoring other individuals passing through the target area.

In addition to behavioural observations, MOSMOKE included an environmental audit of tobacco and vape-related litter. This audit recorded the presence of the following items: cigarette butts, packaging, rolling paper/filter waste, lighters, vape pens, cartridges, pods and stickers. Observers arrived at least 15 min before each observation period to complete this environmental audit.

A detailed instruction manual for using MOSMOKE is provided in online supplemental material 1. The observation form and data summary sheet are available in onlinesupplemental material 2  3, respectively.

### Inter-rater reliability

High inter-rater reliability (ie, consistent agreement between observers) is essential for ensuring the validity of any psychometric tool. Without it, a tool cannot be considered a reliable measure of the behaviours it aims to capture. To assess the inter-rater reliability of MOSMOKE, three observers (JSB, SB and AK) independently conducted observations during 12 5 min periods on a weekday (Tuesday) in February 2024. All three observers used the MOSMOKE tool simultaneously within the same target area.

The lead researcher (JSB) trained the other two observers (SB and AK). JSB has over 400 hours of experience conducting systematic observations with the MOHAWk tool and was involved in the development and piloting of MOSMOKE. SB contributed to MOSMOKE’s development and piloting but had no prior experience with systematic observation tools. AK had no previous experience with either MOSMOKE or other systematic observation tools.

Training consisted of reading the MOSMOKE instruction manual (see online supplemental material 1), followed by supervised practice at the study site. During practice sessions, the observers reviewed and resolved discrepancies through discussion. In total, training and practice took approximately 8 hours.

### Main study

This study is reported in accordance with the Strengthening the Reporting of Observational Studies in Epidemiology cross-sectional guidelines^33^ (see online supplemental material 4).

#### Study design

This was an observational study using the MOSMOKE tool to collect data over 4 weekdays (Friday, Monday, Tuesday and Friday) in March 2024. Observations took place at four different sites within St Peter’s Square. These sites were selected based on two key environmental factors, creating a 2×2 factorial design to assess the criterion-related validity of MOSMOKE—that is, how well the MOSMOKE tool’s measurements predict or correlate with an external criterion.

#### Study sites

**Figure 1:**
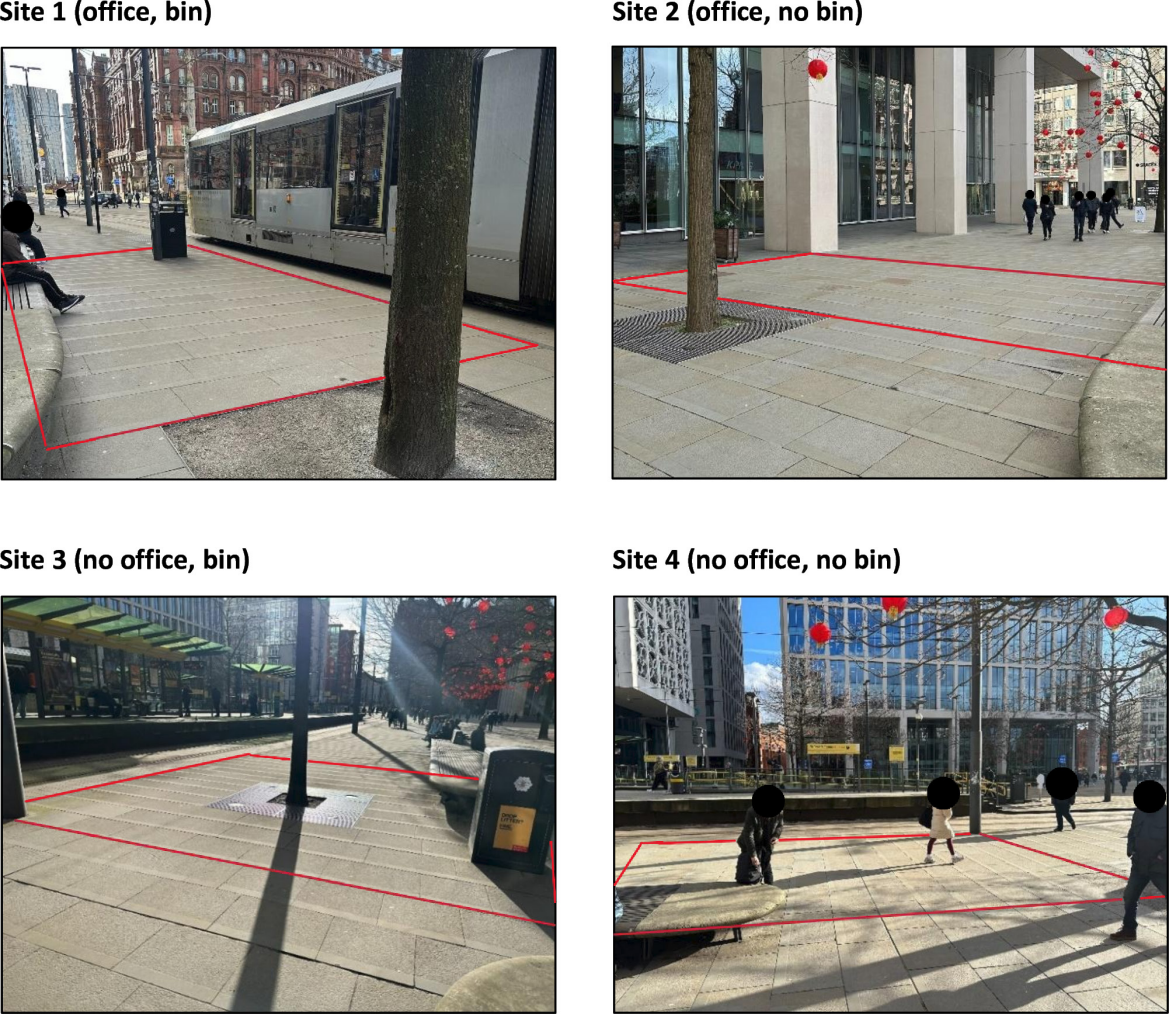
Photographs of the four study sites (target area boundaries outlined in red).

Criterion-related validity was assessed by examining two environmental factors hypothesised to influence smoking behaviours: the presence of a smoking bin, which may prompt smoking by providing disposal options, and adjacency to office block entrances, which may attract smokers on breaks due to indoor smoking restrictions in the United Kingdom (UK).^34^ Accordingly, four sites within St Peter’s Square were selected based on (1) presence of a smoking bin (bin vs no bin) and (2) adjacency to an office block (office vs no office) (see figure 1). Criterion-related validity would be supported if smoking behaviours were higher near smoking bins and office entrances. In contrast, no significant differences were hypothesised for vaping behaviours because vape products are not typically discarded in bins, and vaping is not restricted indoors under UK smoke-free legislation.^34^

#### Observation schedule

Observations occurred during four 1-hour periods each day: 10:30–11:30, 12:00–13:00, 14:00–15:00 and 15:30–16:30. Previous research indicates that shortened observation schedules (eg, 2 days with four observation periods per day) can provide reliable estimates of activity in public spaces.^26^ In total, 32 observation hours were completed (8 hours per site). Observation periods were counterbalanced by day of the week and time of day to ensure balanced rotation between paired sites (see online supplemental material 5 for details).

#### Analyses

The unit of analysis was at the level of the observation period, that is, counts per observation period. All data were analysed using SPSS V.28.

##### Inter-rater reliability

Inter-rater reliability between each pair of observers was assessed using two-way mixed, single measure, consistency intraclass correlation coefficients (ICCs). ICCs can be interpreted as <0.5=‘poor‘; 0.5–0.75=‘moderate’; 0.76–0.9=‘good’ and >0.9=‘excellent’.^35^

##### Criterion-related validity

Criterion-related validity was assessed using Mann-Whitney U tests to compare smoking and vaping behaviours across sites, based on the presence of a smoking bin (bin vs no bin) and adjacency to an office (office vs no office).

### Patient and public involvement

Patients or the public were not involved in the design, conduct, reporting or dissemination plans of this research.

## Results

### Inter-rater reliability

Table 1 shows the inter-rater reliability (ICCs) for all three pairs of observers across all MOSMOKE coding categories. Inter-rater reliability was ‘good’ (ICCs>0.75; n=4) or ‘excellent’ (ICCs>0.90; n=2) for coding smoking behaviours (ICCs=0.78–1) and mostly ‘good’ (n=4) or ‘excellent’ (n=1) for coding vaping behaviours (ICCs=0.73–1) (table 1). Inter-rater reliability was slightly lower for coding individuals inhaling a cigarette or vape, compared with those holding a cigarette or vape (table 1).

**Table 1:** Inter-rater reliability for each pair of observers

MOSMOKE code		ICCs (95% CI)		
		Observer pair 1: SB, AK	Observer pair 2: SB, JSB	Observer pair 3: JSB, AK
Smoking	Holding cigarette	1* (1.00 to 1.00)	0.81† (0.47-0.94)	0.81† (0.47-0.94)
	Inhaling cigarette	1* (1.00 to 1.00)	0.78† (0.37-0.93)	0.78† (0.37-0.93)
Vaping	Holding vape	0.87† (0.62-0.96)	0.87† (0.62-0.96)	0.88† (0.63-0.96)
	Inhaling vape	0.88† (0.64-0.96)	0.90† (0.70-0.97)	0.73 (0.29-0.91)
Total number of people		0.98* (0.94-0.995)	0.99* (0.96-0.997)	0.98* (0.92-0.99)
Age group	Infant	1* (1.00 to 1.00)	0.40 (−0.20-0.78)	0.40 (−0.20-0.78)
	Child	0.98* (0.94-0.995)	0.98* (0.94-0.995)	0.96* (0.88-0.99)
	Teen	0.53 (−0.03-0.84)	0.51 (−0.04-0.83)	0.35 (−0.25-0.76)
	Adult	1* (1.00 to 1.00)	0.99* (0.96-0.996)	0.97* (0.89-0.99)
	Older adult	0.85† (0.55-0.95)	0.99* (0.95-0.996)	.89† (0.67-0.97)

* ICC>0.90=‘excellent’ reliability.

† ICC>0.75=‘good’ reliability.

ICCs, intraclass correlation coefficients; MOSMOKE, Method for Observing SMOKing and vaping bEhaviours.

For coding age groups, inter-rater reliability varied. Inter-rater reliability was ‘good’ to ‘excellent’ for children (ICCs=0.96–0.98), adults (ICCs=0.97–1) and older adults (ICCs=0.85–0.99) (table 1). Inter-rater reliability was lower for infants (ICCs=0.40–1) and teens (ICCs=0.35–0.53), with ICCs ranging from ‘poor’ to ‘excellent’ (table 1).

## Main study

Table 2 summarises the descriptive statistics for the main study. Precipitation occurred during 4 of the 32 observation periods. Across the four sites, a total of 14 980 individuals were observed during 32 hours of data collection. Observers coded more individuals holding a vape (n=570) than holding a cigarette (n=345).

**Table 2:** Smoking and vaping behaviours and sample characteristics across all four sites

Site	Total hours	Total number of people observed	Smoking(% of total people)		Vaping(% of total people)		Age group (% of total people)				
			Holding cigarette	Inhaling cigarette	Holding vape	Inhaling vape	Infant	Child	Teen	Adult	Older adult
Site 1(office, bin)	8 hours	4614*M: 576.8SD: 162.3*	152 (3.3%)*M: 19.0SD: 5.3*	89 (1.9%)*M: 11.1SD: 4.4*	144 (3.1%)*M: 18.0SD: 8.8*	58 (1.3%)*M: 7.3SD: 3.5*	20(0.4%)*M: 2.5SD: 1.1*	121 (2.6%)*M: 15.1SD: 21.4*	233 (5.1%)*M: 29.1SD: 37.8*	4100 (88.9%)*M: 512.5SD: 114.9*	143 (3.1%)*M: 17.9SD: 13.6*
Site 2(office, no bin)	8 hours	4112*M: 514.0SD: 164.1*	60 (1.5%)*M: 7.5SD: 4.8*	42 (1.0%)*M: 5.3SD: 3.0*	174(4.2%)*M: 21.8SD: 11.3*	70(1.7%)*M: 8.8SD: 5.0*	24(0.6%)*M: 3.0SD: 2.4*	17(0.4%)*M: 2.1SD: 2.7*	52(1.3%)*M: 6.5SD: 6.7*	3945 (95.9%)*M: 493.1SD: 160.9*	74(1.8%)*M: 9.3SD: 8.5*
Site 3(no office, bin)	8 hours	2622*M: 327.8SD: 136.6*	52 (2.0%)*M: 6.5SD: 4.3*	25 (1.0%)*M: 3.1SD: 2.7*	110 (4.2%)*M: 13.8SD: 10.2*	34 (1.3%)*M: 4.3SD: 3.1*	13(0.5%)*M: 1.6SD: 0.7*	9(0.3%)*M: 1.1SD: 1.2*	258 (9.8%)*M: 32.3SD: 68.8*	2255 (86.0%)*M: 281.9SD: 89.0*	87(3.3%)*M: 10.9SD: 3.0*
Site 4(no office, no bin)	8 hours	3632*M: 454.0SD: 194.9*	81 (2.2%)*M: 10.1SD: 4.0*	39 (1.1%)*M: 4.9SD: 2.2*	142 (3.9%)*M: 17.8SD: 7.5*	44 (1.2%)*M: 5.5SD: 4.2*	17(0.5%)*M: 2.1SD: 2.5*	35(1.0%)*M: 4.4SD: 9.0*	344(9.5%)*M: 43.0SD: 79.7*	3152(86.8%)*M: 394.0SD: 139.4*	84(2.3%)*M: 10.5SD: 4.9*
All sites	32 hours	14 980*M: 468.1 SD: 183.2*	345 (2.3%)*M: 10.8 SD: 6.7*	195 (1.3%)*M: 6.1 SD: 4.3*	570 (3.8%)*M: 17.8 SD: 9.5*	206 (1.4%)*M: 6.4 SD: 4.2*	74(0.5%)*M: 2.3 SD: 1.8*	182 (1.2%)*M: 5.7 SD: 12.5*	887 (5.9%)*M: 27.7 SD: 54.9*	13 452 (89.8%)*M: 420.4 SD: 153.9*	388 (2.6%)*M: 12.1 SD: 8.8*

M, Mean; SD, Standard Deviation.

Table 3 shows smoking and vaping behaviour by age group. Adults made up the vast majority of observed individuals (n=13 452; 89.8%). Among adults, 2.3% (n=345) were holding a cigarette and 3.8% (n=570) were holding a vape. Among teens, no individuals were observed smoking, but 5.0% (n=44) were holding a vape. A small proportion of older adults were holding cigarettes (2.6%; n=10), with only two older adults observed holding a vape (0.05%).

**Table 3:** Frequency and proportion of individuals smoking and vaping across age groups

Age group	Total hours	Total number of people observed	Smoking (% of total people)		Vaping (% of total people)	
			Holding cigarette	Inhaling cigarette	Holding vape	Inhaling vape
Teen	8 hours	887*M: 27.7SD: 54.9*	0	0	44 (5.0%)*M: 1.38SD: 3.9*	12 (1.4%)*M: 0.38SD: 1.2*
Adult	8 hours	13 452*M: 420.4SD: 153.9*	335 (2.5%)*M: 10.5SD: 6.6*	187 (1.4%)*M: 5.8SD: 4.3*	524 (3.9%)*M: 16.38SD: 8.9*	192 (1.4%)*M: 6.0SD: 4.0*
Older adult	8 hours	388*M: 12.1SD: 8.8*	10 (2.6%)*M: 0.31SD: 0.6*	6 (1.5%)*M: 0.2SD: 0.5*	2 (0.05%)*M: 0.06SD: 0.2*	1 (0.03%)*M: 0.03SD: 0.2*

M, Mean; SD, Standard Deviation.

Litter data indicated that cigarette butts were the most commonly recorded item (n=72), accounting for 92.3% of all recorded litter items. Other smoking-related litter was minimal, including five instances of rolling paper or filter waste and one discarded vape pen or cartridge. No cigarette packaging, lighters or vape-related stickers (on the ground) were recorded.

### Criterion-related validity

There were significantly more smoking behaviours observed at sites with a smoking bin (n=204) compared with sites without a bin (n=141) (U=1564.0, p=0.02, r=0.21). Similarly, more smoking behaviours were observed at office sites (n=212) compared with non-office sites (n=133) (U=1478.00, p=0.006, r=0.24).

In contrast, vaping behaviours did not differ significantly across these site characteristics. There were no significant differences between sites with a smoking bin (n=254) and those without (n=316) (U=1678.50, p=0.08, r=0.16), or between office (n=318) and non-office sites (n=252) (U=1664.0, p=0.07, r=0.25).

## Discussion

### Summary of key findings

This study provides preliminary evidence that MOSMOKE is a reliable and valid tool for assessing smoking and vaping behaviours in public spaces. The tool showed high inter-rater reliability between observers, indicating that MOSMOKE is intuitive and requires minimal training for effective application. Observed smoking and vaping at different sites aligned with the prespecified hypotheses, providing preliminary evidence that the codes used in MOSMOKE are valid.

### Comparison with prior work

MOSMOKE addresses a critical gap in unobtrusive measurement tools for capturing smoking and vaping behaviours in specific environmental contexts. Its ability to detect differences in these behaviours across different sites demonstrates its potential to generate valuable evidence on how environmental factors influence smoking and vaping. This is particularly notable because previous natural experimental studies of smoking and vaping interventions in outdoor public spaces have often relied on self-reported data, which may not be sensitive enough to detect changes in behaviour within the specific environments targeted by these interventions. For example, a natural experimental study in Canada of restricted e-cigarette use in public places found no impact on adults' vaping and smoking behaviours.^36^ However, the study relied on national survey data, which the authors acknowledged was insufficient to evaluate changes in vaping at the specific locations targeted by the bans.

MOSMOKE’s high inter-rater reliability, even with minimal training or prior experience, indicates that it is a user-friendly tool that can be deployed quickly without the need for participant recruitment. This makes MOSMOKE especially useful for collecting crucial baseline data before interventions are implemented, making it ideal for evaluating the before-and-after effects of policies and interventions that can sometimes be implemented at short notice.

Inter-rater reliability for coding age group was generally lower than that reported in observation tools assessing physical activity behaviours that use the same age group categories.^37 38^ This lower reliability may be partly explained by the high footfall at the sites observed in the present study—an average of 468 people per hour—which can make accurate coding more difficult. This finding is important as many studies observing smoking behaviours do not report inter-rater reliability, even when collecting data on multiple variables. For example, a previous study observed smoking behaviours alongside physical activity, alcohol use, age group, gender and social group composition but did not assess inter-rater reliability.^22^ The present findings suggest that tools designed to capture multiple behaviours and participant characteristics simultaneously may not always produce reliable data for smoking and vaping behaviours.

The higher prevalence of vaping among teens (5.0%), compared with no observed cigarette use, reflects the well-documented trend of declining smoking rates and rapidly rising vaping rates among younger populations.^39^ By contrast, the observed prevalence of vaping in older adults was very low (0.05%), supporting prior research that older adults are less likely to use e-cigarettes.^40^ This further supports the criterion-related validity of MOSMOKE and demonstrates its ability to capture age-specific trends in smoking and vaping behaviours across different environmental contexts.

### Strengths and limitations

To the authors’ knowledge, this is the first study to test the reliability and validity of systematically observing smoking and vaping behaviours in public spaces. The study involved three observers across four busy sites at various times throughout the week. MOSMOKE can be used by a single observer with minimal training. We have provided normative data (table 2) to support sample size calculations for natural experimental studies using this tool to evaluate environmental interventions.

However, there are some limitations. The relatively small sample size resulted in wide CIs for some reliability estimates (table 1). Since participant surveys were not conducted, there is a risk that certain behaviours may have been misclassified. For example, heated tobacco products—which use real tobacco but resemble vaping devices—might have been coded as vaping, even though they are more similar to cigarettes due to their use of tobacco. Observers reported rare instances where it was difficult to distinguish vaping devices from other handheld items (eg, mobile phones), but this did not appear to substantially affect inter-rater reliability, which remained ‘good’ for identifying individuals holding a vape across all observer pairs (see table 1). The study was also limited to daylight hours for ethical reasons, so it is unclear if smoking and vaping behaviours would be observed as reliably at night.

Another limitation relates to the coding of estimated age groups, which showed lower inter-rater reliability for the ‘teen’ category. Although the same procedure for coding age group has shown high reliability in previous studies using the MOHAWk tool, reliability was lower here. This may be partly due to fewer contextual cues, as data collection for inter-rater reliability took place during the UK half term when many teens were not wearing school or college uniforms, which can help distinguish them from adults. Additionally, observer training focused more on smoking and vaping behaviours, which might have reduced attention to estimating age. Future studies aiming to assess differences by age group should place greater emphasis on practice and training for estimating age to improve reliability.

Despite these limitations, this initial work has developed a reliable observation measure that provides an important foundation for future research to determine whether these potential limitations pertain.

### Implications for policy and practice

As countries consider policies aimed at reducing smoking and vaping in outdoor settings, it is crucial that these policies are informed by objective, reliable data. An important application of MOSMOKE is to fill a gap in the evidence on the before-and-after impacts of smoking and vaping policies and interventions in outdoor public spaces. Since researchers often do not control the implementation of such policies, natural experiments (ie, real-world interventions outside researchers’ control) are ideal for assessing their causal effects. These studies are particularly valuable as they help policymakers understand what works in real-world settings.^41^ For example, MOSMOKE could be used to assess the effects of changing environmental cues, such as smoking advertisements or the removal of cigarette butt bins.

The tool is also valuable for detecting unintended consequences of smoking policies on vaping behaviours. For example, advertisements promoting vaping as a quitting aid might inadvertently lead to increased vaping among younger people.^12^ Given the large socioeconomic inequalities in smoking,^42^ MOSMOKE has the potential to provide valuable insights into the effects of interventions in socioeconomically deprived areas, as it does not require explicit recruitment of participants. This is an advantage over traditional research, which often struggles to adequately recruit participants from underserved population groups.^43^

### Future research

Ongoing testing and refinement of the MOSMOKE tool is essential to ensure its continued relevance, especially as vaping products and usage patterns evolve. Further validity testing should confirm that observers accurately classify smoking and vaping behaviours. This could be achieved through intercept surveys that compare observers’ coded behaviours with participants’ self-reported behaviour.

Given that one of the main applications of MOSMOKE is to evaluate the effects of environmental interventions, it is important to apply it in natural experimental studies to assess preintervention and postintervention changes. This would provide evidence that MOSMOKE is sensitive to change and capable of detecting intervention effects. Additionally, triangulating MOSMOKE data with other methods—such as surveys, environmental exposure assessment, or qualitative interviews—would provide a more comprehensive understanding of the broader behavioural and environmental impacts. Future research should also test MOSMOKE across a variety of different environments—such as parks, transportation hubs, and areas where vaping is more prevalent—to ensure it accurately captures diverse smoking and vaping behaviours.

With the increasing use of video cameras for systematic observation in public spaces,^44^ including reliable night-time observation,^45^ there is potential to develop a camera-based version of MOSMOKE. This could enable the simultaneous coding of demographic characteristics (eg, gender) and additional smoking-related behaviours, for example, coding people’s proximity to others to assess secondhand smoke exposure, a key target of many outdoor smoking policies.

### Conclusions

This study provides preliminary evidence that MOSMOKE is a reliable and valid tool for systematically observing smoking and vaping behaviours in public spaces. The tool is freely available for use and includes a detailed instruction manual, observation form, data summary form, all provided in onlinesupplemental files 1^3^. MOSMOKE is particularly valuable for unobtrusively assessing how smoking and vaping behaviours vary across different environmental contexts and for evaluating changes in response to policies and interventions.

## Supplementary material

10.1136/bmjopen-2025-105510 online supplemental file 1

10.1136/bmjopen-2025-105510 online supplemental file 2

10.1136/bmjopen-2025-105510 online supplemental file 3

10.1136/bmjopen-2025-105510 online supplemental file 4

10.1136/bmjopen-2025-105510 online supplemental file 5

## Data Availability

Data are available on reasonable request.
