# Supplementary material for: Method for Observing SMOKing and vaping bEhaviours (MOSMOKE): development and validation of a systematic observation tool
Source: BMJ Open. 2025 Jul 13;15(7):e105510. doi: 10.1136/bmjopen-2025-105510 (PMC12258348; doi:10.1136/bmjopen-2025-105510)
Supplement: online supplemental file 1 [file bmjopen-15-7-s001.docx]

**MOSMOKE**

**(Method for Observing SMOking and vaping bEhaviours)**

**Instruction Manual**

Revised 20/05/2025

**CONTENTS**

**Page**

1. [**OVERVIEW OF MOSMOKE 3**](#Overview_of_MOHAWk)
2. [**OBSERVATION PREPARATION 4**](#Observation_preparation)
3. [**CODES AND RECORDING 5**](#Codes_and_recording)
4. [**OBSERVATION PROCEDURES**](#Observation_procedures)  **7**
5. [**OBSERVATION PERIODS**](#Observation_periods)  **9**
6. [**DATA ANALYSIS 9**](#Data_analysis)

[**APPENDIX 1 – MOSMOKE observation form 1**](#Appendix_1)**1**

[**APPENDIX 2 – MOSMOKE data summary form 2**](#Appendix_2)**0**

[**APPENDIX 3 – Example of a completed MOSMOKE observation form**](#Appendix_1) **22**

[**APPENDIX 4 – Example of a completed MOSMOKE data summary form 2**](#Appendix_2)**3**

1. **OVERVIEW OF MOSMOKE**

**Introduction to MOSMOKE**

MOSMOKE (Method for Observing SMOking and vaping bEhaviours) is a systematic observation tool designed for assessing smoking and vaping behaviours in public spaces. The tool focuses on recording the number of individuals who enter a designated ‘target area’, categorising their age group, and identifying whether they engage in smoking or vaping behaviours. Additionally, it includes an environmental audit of smoking and vaping-related litter. MOSMOKE is versatile and can be applied to a wide variety of public spaces including urban parks, public squares, and other outdoor spaces.

MOSMOKE is freely available and includes an instruction manual, a standardised observation form, and a summary form. Further information and guidance are available in a peer-reviewed journal article, with all forms provided in the appendices.

**Summary of how MOSMOKE works**

MOSMOKE observations are conducted by continuously scanning a designated ‘target area’ over a defined observation period. Data are recorded manually using pen and paper, with a focus on two main components:

1. ***Behaviour observations***

Observers classify individuals entering the target area by age group and record any visible smoking or vaping behaviour:

- Age group classification
  - Infant
  - Child
  - Teen
  - Adult
  - Older Adult
- Smoking
  - Holding a cigarette
  - Inhaling a cigarette (arm to mouth)
- Vaping
  - Holding a vape
  - Inhaling from a vape (arm to mouth)

1. ***Environmental audit (conducted before observations)***

Observers record the presence of smoking- and vaping-related litter within the target area prior to beginning behaviour observations. This includes:

- Cigarette butts
- Cigarette packaging / tobacco pouches
- Rolling paper and filter waste
- Lighters
- Vape pens / cartridges / pods
- Vape stickers

1. **OBSERVATION PREPARATION**

***Defining the target area***

Before beginning data collection, all observers must visit the site together to agree on the precise boundaries of the target area. This is important so that all observers can agree on whether an individual falls within the boundary of that area or not.

Only individuals within the target area should be recorded. While behaviours outside the area (e.g., someone holding a vape before entering) may be visible, they should only be recorded once the person enters the defined target space. However, such observations can help prepare the observer to record accurately once the individual enters.

A target area should be large enough to capture meaningful activity, but small enough for observers to clearly see and reliably record all individuals and their behaviours. It should be placed where smoking or vaping activity is likely to occur. The target area should be small enough so that observers can reliably view and record the characteristics and activity of all people that enter the target area.

Observers should identify a designated observation position that offers a clear, unobstructed view of the target area and, where possible, provides cover from adverse weather. If an area that affords a good view with shelter is not possible, an area that affords a reasonable view with good cover should be identified for particularly severe weather. This is important because observations are carried out regardless of weather conditions, unless weather conditions become so extreme that they compromise the observer’s safety.

The boundaries of the target area do not need to be physically marked, but using natural markers like fences, roadways, or pavement markings can help define the space. The same boundaries must be used across all observation periods.

At least one “safe” area should be identified, where the observer can go to if they feel unsafe or encounter aggression from the public (e.g., café, public library).

***Observer training***

All observers must be fully trained in how to use MOSMOKE before formal data collection begins. Ideally, training should be conducted jointly, allowing observers to complete practice observations together. This will help calibrate observers and allow observers to agree on any ambiguities, thereby improving agreement between observers i.e. ‘inter-rater reliability’.

To formally assess agreement between observers, inter-rater reliability should be calculated using intraclass correlation coefficients (ICC). Observers should aim for an ICC of at least 0.75, which indicates acceptable reliability. High inter-rater reliability ensures that data collected by multiple observers using MOSMOKE are consistent and comparable. If discrepancies in observations arise, they should be discussed and resolved collectively during training or calibration sessions.

***Before observations***

Before starting the observation session, observers should print out a copy of the site map to clearly define the target area. This helps ensure all observers have a shared understanding of the exact boundaries for consistent data collection. Observers should also bring several copies of an information letter about the study, which includes details such as the purpose of the research and ethical approval. This letter can be provided to any member of the public who asks about the observers’ activities.

Observers should arrive at the site at least 15 minutes before the official observation period starts. During this time, they should record the frequency of smoking- and vaping-related litter in the target area, including cigarette butts, packaging, rolling paper and filter waste, lighters, vape pens or cartridges, and vape stickers – see next section. Taking photographs can also help document the physical environment.

1. **CODES AND RECORDING**

**Smoking and vaping litter environmental audit**

The litter audit should be conducted before the observation period begins. The following items should be counted (using the MOSMOKE data summary form):

| **Litter item** | **Definition** |
| --- | --- |
| **Cigarette butts** | Remnants of cigarettes, including the filter, left behind after smoking. |
| **Cigarette packaging / tobacco pouches** | Packaging materials from cigarettes or tobacco, including empty cigarette packs or tobacco pouches. |
| **Rolling paper and filter waste** | Leftover rolling papers and filters used for hand-rolled cigarettes. |
| **Lighters** | Disposable or reusable devices used to ignite cigarettes. |
| **Vape pens / cartridges / pods** | Discarded vape devices, cartridges, or pods, typically containing e-liquid for e-cigarettes. |
| **Vape stickers** | Promotional or branded stickers from vape pens or e-cigarettes. |

**MOSMOKE observations**

***Age group***

Infant = Babies or toddlers in a pram, sling, or other baby carrier.

NOTE: Do not record any behaviours or demographic characteristics for infants.

Child = Individuals who appear to be aged from infancy to 12 years of age.

NOTE: Children can often be identified by their general appearance, such as wearing a school uniform, and they are more likely to be accompanied by a parent or guardian.

Teen = Individuals who appear to be between 13 and 19 years of age

NOTE: This category includes anyone who looks like a secondary school student, college student, or university undergraduate.

Adult = Individuals who appear to be between 20 to 74 years of age.

Older Adult = Individuals who appear to be 75 years of age and older.

NOTE: Observers should identify older adults based on general appearance and mobility. Key indicators include gait and movement patterns that might be stiffer, slower, or otherwise restricted. Physical signs of aging such as grey hair, wrinkles, or hair loss should also be considered to distinguish age-related changes from other impairments. If a person is wearing a work-related uniform, they are more likely to be classified as an adult rather than an older adult.

***Smoking***

Holding cigarette = Record when an individual is visibly holding a cigarette, regardless of whether they are actively smoking it or not.

NOTE: This includes individuals with cigarettes in their hands or between their fingers. If unsure, then do not record it as ‘holding a cigarette’.

Inhaling a cigarette = Record when an individual brings a cigarette to their mouth.

NOTE: Include instances where the cigarette is placed between their lips when lighting it or holding it in their mouth. Visible signs of inhalation, such as exhaling smoke, can help confirm use but are not required for recording.

***Vaping***

Holding a vape = Record when an individual is visibly holding a vape device (including pens, pods, or e-cigarettes).

NOTE: Be mindful of the size and shape of vapes to avoid confusion with other objects. If unsure, do not record it as ‘holding a vape.’

Inhaling from a vape = Record when an individual brings a vape device to their mouth.

NOTE: As with cigarette use, signs of inhalation such as exhaling vapor can help confirm use but are not required for recording. Be aware that vapor may be less visible than cigarette smoke.

***Group***

This column is intended to help observers efficiently record the number of individuals when observing smaller groups. However, observers should not record large groups of 10 or more people together, as doing so can reduce the accuracy of coding smoking and vaping behaviours.

**Notes**

A column is provided for observers to make notes on individual people when necessary. This is especially useful to keep track of individuals who remain in the target area for a prolonged period, helping to avoid double counting them and their activities. See Appendix 3 for an example.

***Weather***

Throughout the observation period, record the approximate start and end times of any precipitation. For example, write down “rain from 10:00-10:15am and 10:40-10:55am”. This should be updated continuously as weather conditions change.

***Comments***

At the top of the observation form, note any other relevant observations during the session. This could include if anyone spoke to the observer, if certain activities were particularly popular, reasons for any missing data, or breaks taken by the observer.

1. **OBSERVATION PROCEDURES**

***How to observe***

Observers should continuously scan the target area throughout the entire observation period. Whenever a person enters the target area, the observer must record their age group (Infant, Child, Teen, Adult, or Older Adult) and, if applicable, any smoking or vaping behaviours.

Record all individuals who enter the target area, except those who are inside vehicles such as cars or motorbikes. If a person leaves and then re-enters the target area during the same observation period, do not record them a second time.

The unit of coding is the behaviour. This means observers should count the number of people performing each behaviour. The same person can be recorded for multiple different behaviours, but each behaviour should only be recorded once per person within the observation period. For example, if someone inhales two cigarettes during the observation period, it should still be recorded as one instance of ‘inhaling a cigarette’ for that individual.

Observers may choose to divide the hour-long observation period into smaller blocks (such as 15-minute intervals) by marking the observation form accordingly (see Appendix 3 for an example). This allows researchers to analyze data by shorter time blocks as well as for the full hour.

If necessary, observers can move around within the target area to improve visibility, as long as they do not interfere with activities and only record individuals who enter the target area.

It may be more challenging to estimate age groups accurately during adverse weather conditions (e.g., when people wear hooded jackets or other protective clothing). In such cases, observers should do their best to estimate the individual’s age group to avoid missing data.

***Observation procedure***

1. Before starting the observation period, record the date, day, site name, observer initials, start time, and end time at the top of the observation form.
2. Conduct an environmental litter audit of the target area using the MOSMOKE data summary form (see Appendix 2 for the form and Appendix 4 for an example).
3. Record the age group and any smoking or vaping behaviours of all individuals entering the target area using the MOSMOKE observation form (see Appendix 1 for the form and Appendix 3 for an example). Also, record the duration of any precipitation during the observation.
4. After the observation period ends, record total counts for each age group and smoking/vaping behaviour on the MOSMOKE data summary form. Include the total duration of any precipitation that occurred.

***Busy periods***

During very busy periods, if it becomes too difficult to record each individual on a separate row, observers should instead record the total number of individuals in each age group and behaviour category in the appropriate boxes. For example, if it is hard to track individual behaviours, simply note the total number of people holding cigarettes or vaping. Appendix 3 includes an example of how to record totals during busy times.

If this approach still does not allow reliable data collection, it may suggest that MOSMOKE is not suitable for that site or time. In such cases, consider reducing the size of the target area or using an alternative observation tool that may be more appropriate.

***Toilet breaks***

A designated location for toilet breaks should be identified during the preparation phase. Ideally, breaks should be taken before or after observation periods. If a break is necessary during observations, it must be documented in the comments section of the MOSMOKE data collection form.

***Respecting the public***

Observers should aim to blend in but remain clearly identifiable (e.g., by wearing a high-visibility vest and using a clipboard). They should be prepared to answer any questions honestly from members of the public during observations.

Observers must always be polite and respectful. If members of the public, especially local residents, request that observations stop, the observer must immediately cease observations and postpone any future observations at that site.

1. **OBSERVATION PERIODS**

***Timing and frequency of observation periods***

Observation periods are one hour long. They can be conducted at any time of day depending on the aims and requirements of the evaluation, as well as available time and resources. For example, MOSMOKE could be used at four set observation periods per day: morning (10-11am), lunchtime (12-1pm), afternoon (3-4pm), and evening (5-6pm).

***Missing or postponed observation periods***

If an observation period is missed (e.g., due to illness) it should be rescheduled for the same time on the next available day in the following week. For example, if a 3-4pm observation period is missed on Monday, it should be made up on the next available Monday at 3-4pm.

***Example observation period***

9:45am - Check target area, prepare data forms and conduct the environmental litter audit in the target area

10am - Start observing in the target area

11am - Stop observing in the target area and transfer data from the MOSMOKE observation form onto the MOSMOKE data summary form

1. **DATA ANALYSIS**

***Summarising data***

After data collection, observers use the MOSMOKE data summary form to total the counts of individuals by age group and their smoking or vaping behaviours. It is important to review the data for completeness and accuracy before proceeding to analysis. Any discrepancies or missing information should be resolved to ensure data quality.

***Sensitivity analysis***

MOSMOKE observations are expected to continue regardless of weather conditions unless the weather becomes so severe that it threatens the safety of the observer. Since weather, particularly precipitation, may influence behaviours in public spaces, it is recommended to perform a sensitivity analysis to assess its impact. Observers record the duration of any precipitation during the observation period. For the sensitivity analysis, observation periods during which precipitation occurs for 50% or more of the time (i.e., 30 minutes or longer) should be excluded from the main analysis. Alternatively, precipitation can be included as a co-variate in the data analysis to control for their effect.

**APPENDIX 1 – MOSMOKE observation form**

**DATE / DAY: ________________________________________ SITE: __________________________________________ OBSERVER: ______________**

**WEATHER / COMMENTS: __**__**____________________________________________________________________________________________________**

*Include the duration of any precipitation e.g. ‘Rain from 10.20-10.45am. Also make a note of any other potentially important observations.*

| **Person** | **Age group** | | | | | **Cigarette smoking** | | **Vaping** | | **Group**  **(record head count in group)** | **Notes** |
| --- | --- | --- | --- | --- | --- | --- | --- | --- | --- | --- | --- |
|  | **Infant** | **Child** | **Teen** | **Adult** | **Older Adult** | **Holding cigarette** | **Inhaling cigarette** | **Holding vape** | **Inhaling from a vape** |  |  |
| 1 |  |  |  |  |  |  |  |  |  |  |  |
| 2 |  |  |  |  |  |  |  |  |  |  |  |
| 3 |  |  |  |  |  |  |  |  |  |  |  |
| 4 |  |  |  |  |  |  |  |  |  |  |  |
| 5 |  |  |  |  |  |  |  |  |  |  |  |
| 6 |  |  |  |  |  |  |  |  |  |  |  |
| 7 |  |  |  |  |  |  |  |  |  |  |  |
| 8 |  |  |  |  |  |  |  |  |  |  |  |
| 9 |  |  |  |  |  |  |  |  |  |  |  |
| 10 |  |  |  |  |  |  |  |  |  |  |  |
| 11 |  |  |  |  |  |  |  |  |  |  |  |
| 12 |  |  |  |  |  |  |  |  |  |  |  |
| 13 |  |  |  |  |  |  |  |  |  |  |  |
| 14 |  |  |  |  |  |  |  |  |  |  |  |
| 15 |  |  |  |  |  |  |  |  |  |  |  |
| 16 |  |  |  |  |  |  |  |  |  |  |  |
| 17 |  |  |  |  |  |  |  |  |  |  |  |
| 18 |  |  |  |  |  |  |  |  |  |  |  |
| 19 |  |  |  |  |  |  |  |  |  |  |  |
| 20 |  |  |  |  |  |  |  |  |  |  |  |
| 21 |  |  |  |  |  |  |  |  |  |  |  |
| 22 |  |  |  |  |  |  |  |  |  |  |  |
| 23 |  |  |  |  |  |  |  |  |  |  |  |
| 24 |  |  |  |  |  |  |  |  |  |  |  |
| 25 |  |  |  |  |  |  |  |  |  |  |  |
| 26 |  |  |  |  |  |  |  |  |  |  |  |
| 27 |  |  |  |  |  |  |  |  |  |  |  |
| 28 |  |  |  |  |  |  |  |  |  |  |  |
| 29 |  |  |  |  |  |  |  |  |  |  |  |
| 30 |  |  |  |  |  |  |  |  |  |  |  |
| 31 |  |  |  |  |  |  |  |  |  |  |  |
| 32 |  |  |  |  |  |  |  |  |  |  |  |
| 33 |  |  |  |  |  |  |  |  |  |  |  |
| 34 |  |  |  |  |  |  |  |  |  |  |  |
| 35 |  |  |  |  |  |  |  |  |  |  |  |
| 36 |  |  |  |  |  |  |  |  |  |  |  |
| 37 |  |  |  |  |  |  |  |  |  |  |  |
| 38 |  |  |  |  |  |  |  |  |  |  |  |
| 39 |  |  |  |  |  |  |  |  |  |  |  |
| 40 |  |  |  |  |  |  |  |  |  |  |  |
| 41 |  |  |  |  |  |  |  |  |  |  |  |
| 42 |  |  |  |  |  |  |  |  |  |  |  |
| 43 |  |  |  |  |  |  |  |  |  |  |  |
| 44 |  |  |  |  |  |  |  |  |  |  |  |
| 45 |  |  |  |  |  |  |  |  |  |  |  |
| 46 |  |  |  |  |  |  |  |  |  |  |  |
| 47 |  |  |  |  |  |  |  |  |  |  |  |
| 48 |  |  |  |  |  |  |  |  |  |  |  |
| 49 |  |  |  |  |  |  |  |  |  |  |  |
| 50 |  |  |  |  |  |  |  |  |  |  |  |
| 51 |  |  |  |  |  |  |  |  |  |  |  |
| 52 |  |  |  |  |  |  |  |  |  |  |  |
| 53 |  |  |  |  |  |  |  |  |  |  |  |
| 54 |  |  |  |  |  |  |  |  |  |  |  |
| 55 |  |  |  |  |  |  |  |  |  |  |  |
| 56 |  |  |  |  |  |  |  |  |  |  |  |
| 57 |  |  |  |  |  |  |  |  |  |  |  |
| 58 |  |  |  |  |  |  |  |  |  |  |  |
| 59 |  |  |  |  |  |  |  |  |  |  |  |
| 60 |  |  |  |  |  |  |  |  |  |  |  |
| 61 |  |  |  |  |  |  |  |  |  |  |  |
| 62 |  |  |  |  |  |  |  |  |  |  |  |
| 63 |  |  |  |  |  |  |  |  |  |  |  |
| 64 |  |  |  |  |  |  |  |  |  |  |  |
| 65 |  |  |  |  |  |  |  |  |  |  |  |
| 66 |  |  |  |  |  |  |  |  |  |  |  |
| 67 |  |  |  |  |  |  |  |  |  |  |  |
| 68 |  |  |  |  |  |  |  |  |  |  |  |
| 69 |  |  |  |  |  |  |  |  |  |  |  |
| 70 |  |  |  |  |  |  |  |  |  |  |  |
| 71 |  |  |  |  |  |  |  |  |  |  |  |
| 72 |  |  |  |  |  |  |  |  |  |  |  |
| 73 |  |  |  |  |  |  |  |  |  |  |  |
| 74 |  |  |  |  |  |  |  |  |  |  |  |
| 75 |  |  |  |  |  |  |  |  |  |  |  |
| 76 |  |  |  |  |  |  |  |  |  |  |  |
| 77 |  |  |  |  |  |  |  |  |  |  |  |
| 78 |  |  |  |  |  |  |  |  |  |  |  |
| 79 |  |  |  |  |  |  |  |  |  |  |  |
| 80 |  |  |  |  |  |  |  |  |  |  |  |
| 81 |  |  |  |  |  |  |  |  |  |  |  |
| 82 |  |  |  |  |  |  |  |  |  |  |  |
| 83 |  |  |  |  |  |  |  |  |  |  |  |
| 84 |  |  |  |  |  |  |  |  |  |  |  |
| 85 |  |  |  |  |  |  |  |  |  |  |  |
| 86 |  |  |  |  |  |  |  |  |  |  |  |
| 87 |  |  |  |  |  |  |  |  |  |  |  |
| 88 |  |  |  |  |  |  |  |  |  |  |  |
| 89 |  |  |  |  |  |  |  |  |  |  |  |
| 90 |  |  |  |  |  |  |  |  |  |  |  |
| 91 |  |  |  |  |  |  |  |  |  |  |  |
| 92 |  |  |  |  |  |  |  |  |  |  |  |
| 93 |  |  |  |  |  |  |  |  |  |  |  |
| 94 |  |  |  |  |  |  |  |  |  |  |  |
| 95 |  |  |  |  |  |  |  |  |  |  |  |
| 96 |  |  |  |  |  |  |  |  |  |  |  |
| 97 |  |  |  |  |  |  |  |  |  |  |  |
| 98 |  |  |  |  |  |  |  |  |  |  |  |
| 99 |  |  |  |  |  |  |  |  |  |  |  |
| 100 |  |  |  |  |  |  |  |  |  |  |  |
| 101 |  |  |  |  |  |  |  |  |  |  |  |
| 102 |  |  |  |  |  |  |  |  |  |  |  |
| 103 |  |  |  |  |  |  |  |  |  |  |  |
| 104 |  |  |  |  |  |  |  |  |  |  |  |
| 105 |  |  |  |  |  |  |  |  |  |  |  |
| 106 |  |  |  |  |  |  |  |  |  |  |  |
| 107 |  |  |  |  |  |  |  |  |  |  |  |
| 108 |  |  |  |  |  |  |  |  |  |  |  |
| 109 |  |  |  |  |  |  |  |  |  |  |  |
| 110 |  |  |  |  |  |  |  |  |  |  |  |
| 111 |  |  |  |  |  |  |  |  |  |  |  |
| 112 |  |  |  |  |  |  |  |  |  |  |  |
| 113 |  |  |  |  |  |  |  |  |  |  |  |
| 114 |  |  |  |  |  |  |  |  |  |  |  |
| 115 |  |  |  |  |  |  |  |  |  |  |  |
| 116 |  |  |  |  |  |  |  |  |  |  |  |
| 117 |  |  |  |  |  |  |  |  |  |  |  |
| 118 |  |  |  |  |  |  |  |  |  |  |  |
| 119 |  |  |  |  |  |  |  |  |  |  |  |
| 120 |  |  |  |  |  |  |  |  |  |  |  |
| 121 |  |  |  |  |  |  |  |  |  |  |  |
| 122 |  |  |  |  |  |  |  |  |  |  |  |
| 123 |  |  |  |  |  |  |  |  |  |  |  |
| 124 |  |  |  |  |  |  |  |  |  |  |  |
| 125 |  |  |  |  |  |  |  |  |  |  |  |
| 126 |  |  |  |  |  |  |  |  |  |  |  |
| 127 |  |  |  |  |  |  |  |  |  |  |  |
| 128 |  |  |  |  |  |  |  |  |  |  |  |
| 129 |  |  |  |  |  |  |  |  |  |  |  |
| 130 |  |  |  |  |  |  |  |  |  |  |  |
| 131 |  |  |  |  |  |  |  |  |  |  |  |
| 132 |  |  |  |  |  |  |  |  |  |  |  |
| 133 |  |  |  |  |  |  |  |  |  |  |  |
| 134 |  |  |  |  |  |  |  |  |  |  |  |
| 135 |  |  |  |  |  |  |  |  |  |  |  |
| 136 |  |  |  |  |  |  |  |  |  |  |  |
| 137 |  |  |  |  |  |  |  |  |  |  |  |
| 138 |  |  |  |  |  |  |  |  |  |  |  |
| 139 |  |  |  |  |  |  |  |  |  |  |  |
| 140 |  |  |  |  |  |  |  |  |  |  |  |
| 141 |  |  |  |  |  |  |  |  |  |  |  |
| 142 |  |  |  |  |  |  |  |  |  |  |  |
| 143 |  |  |  |  |  |  |  |  |  |  |  |
| 144 |  |  |  |  |  |  |  |  |  |  |  |
| 145 |  |  |  |  |  |  |  |  |  |  |  |
| 146 |  |  |  |  |  |  |  |  |  |  |  |
| 147 |  |  |  |  |  |  |  |  |  |  |  |
| 148 |  |  |  |  |  |  |  |  |  |  |  |
| 149 |  |  |  |  |  |  |  |  |  |  |  |
| 150 |  |  |  |  |  |  |  |  |  |  |  |
| 151 |  |  |  |  |  |  |  |  |  |  |  |
| 152 |  |  |  |  |  |  |  |  |  |  |  |
| 153 |  |  |  |  |  |  |  |  |  |  |  |
| 154 |  |  |  |  |  |  |  |  |  |  |  |
| 155 |  |  |  |  |  |  |  |  |  |  |  |
| 156 |  |  |  |  |  |  |  |  |  |  |  |
| 157 |  |  |  |  |  |  |  |  |  |  |  |
| 158 |  |  |  |  |  |  |  |  |  |  |  |
| 159 |  |  |  |  |  |  |  |  |  |  |  |
| 160 |  |  |  |  |  |  |  |  |  |  |  |
| 161 |  |  |  |  |  |  |  |  |  |  |  |
| 162 |  |  |  |  |  |  |  |  |  |  |  |
| 163 |  |  |  |  |  |  |  |  |  |  |  |
| 164 |  |  |  |  |  |  |  |  |  |  |  |
| 165 |  |  |  |  |  |  |  |  |  |  |  |
| 166 |  |  |  |  |  |  |  |  |  |  |  |
| 167 |  |  |  |  |  |  |  |  |  |  |  |
| 168 |  |  |  |  |  |  |  |  |  |  |  |
| 169 |  |  |  |  |  |  |  |  |  |  |  |
| 170 |  |  |  |  |  |  |  |  |  |  |  |
| 171 |  |  |  |  |  |  |  |  |  |  |  |
| 172 |  |  |  |  |  |  |  |  |  |  |  |
| 173 |  |  |  |  |  |  |  |  |  |  |  |
| 174 |  |  |  |  |  |  |  |  |  |  |  |
| 175 |  |  |  |  |  |  |  |  |  |  |  |
| 176 |  |  |  |  |  |  |  |  |  |  |  |
| 177 |  |  |  |  |  |  |  |  |  |  |  |
| 178 |  |  |  |  |  |  |  |  |  |  |  |
| 179 |  |  |  |  |  |  |  |  |  |  |  |
| 180 |  |  |  |  |  |  |  |  |  |  |  |
| 181 |  |  |  |  |  |  |  |  |  |  |  |
| 182 |  |  |  |  |  |  |  |  |  |  |  |
| 183 |  |  |  |  |  |  |  |  |  |  |  |
| 184 |  |  |  |  |  |  |  |  |  |  |  |
| 185 |  |  |  |  |  |  |  |  |  |  |  |
| 186 |  |  |  |  |  |  |  |  |  |  |  |
| 187 |  |  |  |  |  |  |  |  |  |  |  |
| 188 |  |  |  |  |  |  |  |  |  |  |  |
| 189 |  |  |  |  |  |  |  |  |  |  |  |
| 190 |  |  |  |  |  |  |  |  |  |  |  |
| 191 |  |  |  |  |  |  |  |  |  |  |  |
| 192 |  |  |  |  |  |  |  |  |  |  |  |
| 193 |  |  |  |  |  |  |  |  |  |  |  |
| 194 |  |  |  |  |  |  |  |  |  |  |  |
| 195 |  |  |  |  |  |  |  |  |  |  |  |
| 196 |  |  |  |  |  |  |  |  |  |  |  |
| 197 |  |  |  |  |  |  |  |  |  |  |  |
| 198 |  |  |  |  |  |  |  |  |  |  |  |
| 199 |  |  |  |  |  |  |  |  |  |  |  |
| 200 |  |  |  |  |  |  |  |  |  |  |  |

**APPENDIX 2 – MOSMOKE data summary form**

**DATE / DAY: ___________________________________ SITE: ____________________________________________ OBSERVER: _______________**

**WEATHER/ COMMENTS:** ______________________________________________________________________________________________________

*Include the duration of any precipitation e.g. ‘Rain from 10.20-10.45am. Also make a note of any other potentially important observations.*

| **AGE GROUP** | **TOTAL COUNT** | **SMOKING** | | **VAPING** | |
| --- | --- | --- | --- | --- | --- |
|  |  | **Holding cigarette** | **Inhaling cigarette** | **Holding vape** | **Inhaling from a vape** |
| **Infant** |  |  |  |  |  |
| **Child** |  |  |  |  |  |
| **Teen** |  |  |  |  |  |
| **Adult** |  |  |  |  |  |
| **Older Adult** |  |  |  |  |  |
| **Group / Busy** |  |  |  |  |  |
|  | **Frequencies by age group**  Infant: Child: Teen: Adult: Older Adult: | | | | |

| **LITTER AUDIT** | **FREQUENCY** |
| --- | --- |
| **Cigarette butts** |  |
| **Cigarette packaging / tobacco pouches** |  |
| **Rolling paper and filter waste** |  |
| **Lighters** |  |
| **Vape pens / cartridges / pods** |  |
| **Vape stickers** |  |

**APPENDIX 3 – Example of a completed MOSMOKE observation form**

**
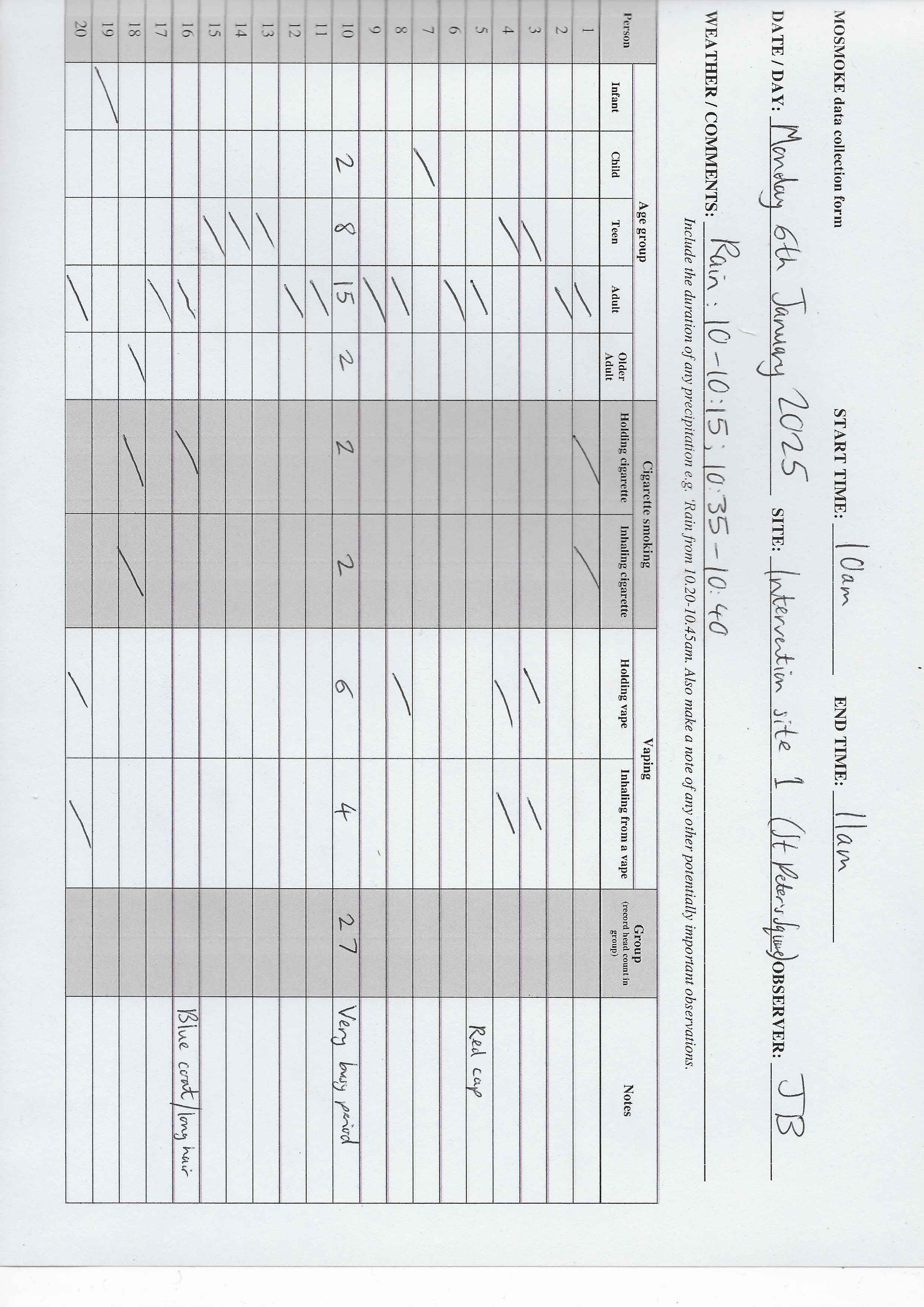
**

**APPENDIX 4 – Example of a completed MOSMOKE data summary form**

**
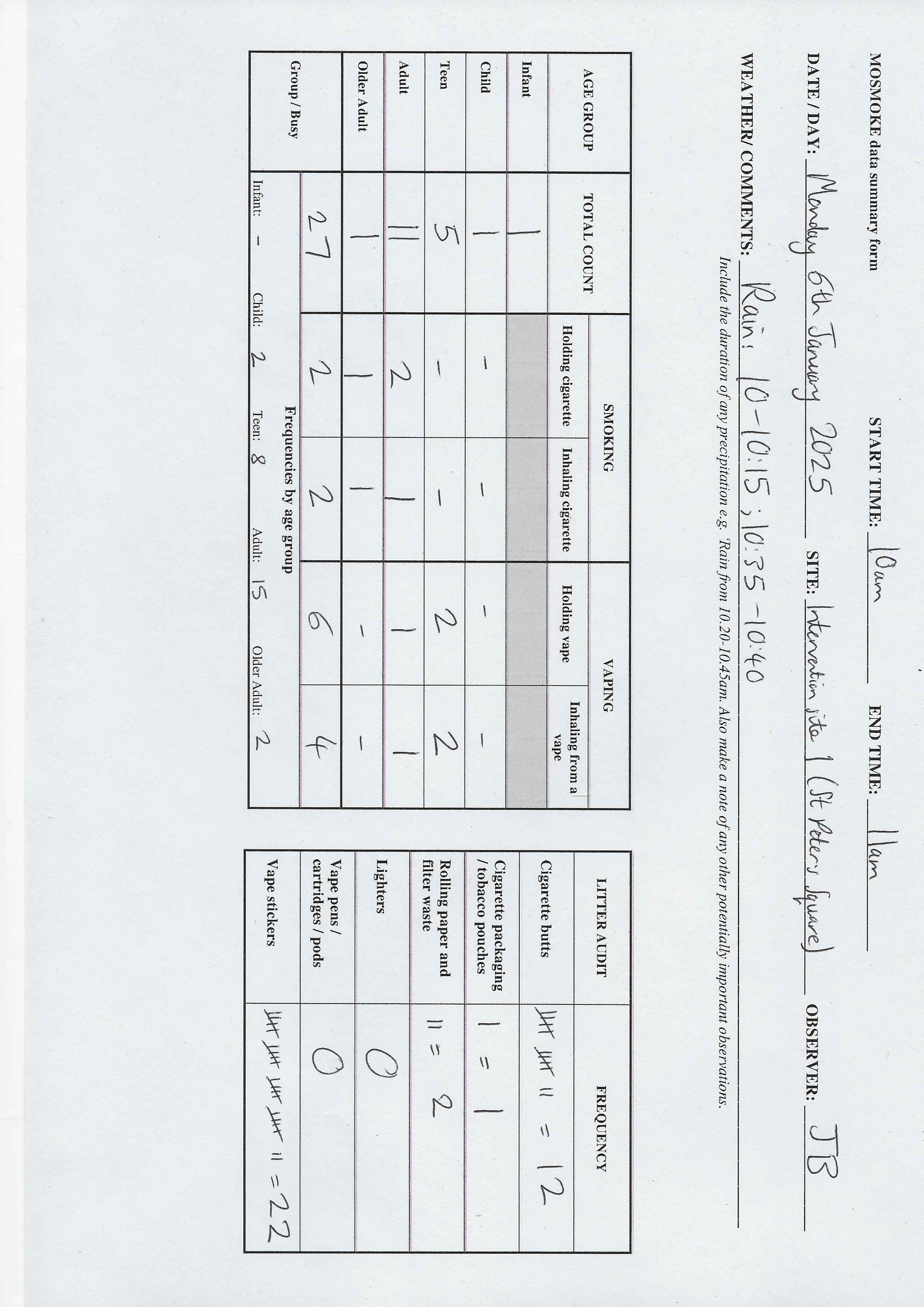
**
