# Supplementary material for: Method for Observing SMOKing and vaping bEhaviours (MOSMOKE): development and validation of a systematic observation tool
Source: BMJ Open. 2025 Jul 13;15(7):e105510. doi: 10.1136/bmjopen-2025-105510 (PMC12258348; doi:10.1136/bmjopen-2025-105510)
Supplement: online supplemental file 2 [file bmjopen-15-7-s002.docx]

**DATE / DAY: _____________________________________________ SITE: _______________________________________________ OBSERVER: ______________**

**WEATHER / COMMENTS: __**__**______________________________________________________________________________________________________________**

*Include the duration of any precipitation e.g. ‘Rain from 10.20-10.45am. Also make a note of any other potentially important observations.*

| **Person** | **Age group** | | | | | **Cigarette smoking** | | **Vaping** | | **Group**  **(record head count in group)** | **Notes** |
| --- | --- | --- | --- | --- | --- | --- | --- | --- | --- | --- | --- |
|  | **Infant** | **Child** | **Teen** | **Adult** | **Older Adult** | **Holding cigarette** | **Inhaling cigarette** | **Holding vape** | **Inhaling from a vape** |  |  |
| 1 |  |  |  |  |  |  |  |  |  |  |  |
| 2 |  |  |  |  |  |  |  |  |  |  |  |
| 3 |  |  |  |  |  |  |  |  |  |  |  |
| 4 |  |  |  |  |  |  |  |  |  |  |  |
| 5 |  |  |  |  |  |  |  |  |  |  |  |
| 6 |  |  |  |  |  |  |  |  |  |  |  |
| 7 |  |  |  |  |  |  |  |  |  |  |  |
| 8 |  |  |  |  |  |  |  |  |  |  |  |
| 9 |  |  |  |  |  |  |  |  |  |  |  |
| 10 |  |  |  |  |  |  |  |  |  |  |  |
| 11 |  |  |  |  |  |  |  |  |  |  |  |
| 12 |  |  |  |  |  |  |  |  |  |  |  |
| 13 |  |  |  |  |  |  |  |  |  |  |  |
| 14 |  |  |  |  |  |  |  |  |  |  |  |
| 15 |  |  |  |  |  |  |  |  |  |  |  |
| 16 |  |  |  |  |  |  |  |  |  |  |  |
| 17 |  |  |  |  |  |  |  |  |  |  |  |
| 18 |  |  |  |  |  |  |  |  |  |  |  |
| 19 |  |  |  |  |  |  |  |  |  |  |  |
| 20 |  |  |  |  |  |  |  |  |  |  |  |
| 21 |  |  |  |  |  |  |  |  |  |  |  |
| 22 |  |  |  |  |  |  |  |  |  |  |  |
| 23 |  |  |  |  |  |  |  |  |  |  |  |
| 24 |  |  |  |  |  |  |  |  |  |  |  |
| 25 |  |  |  |  |  |  |  |  |  |  |  |
| 26 |  |  |  |  |  |  |  |  |  |  |  |
| 27 |  |  |  |  |  |  |  |  |  |  |  |
| 28 |  |  |  |  |  |  |  |  |  |  |  |
| 29 |  |  |  |  |  |  |  |  |  |  |  |
| 30 |  |  |  |  |  |  |  |  |  |  |  |
| 31 |  |  |  |  |  |  |  |  |  |  |  |
| 32 |  |  |  |  |  |  |  |  |  |  |  |
| 33 |  |  |  |  |  |  |  |  |  |  |  |
| 34 |  |  |  |  |  |  |  |  |  |  |  |
| 35 |  |  |  |  |  |  |  |  |  |  |  |
| 36 |  |  |  |  |  |  |  |  |  |  |  |
| 37 |  |  |  |  |  |  |  |  |  |  |  |
| 38 |  |  |  |  |  |  |  |  |  |  |  |
| 39 |  |  |  |  |  |  |  |  |  |  |  |
| 40 |  |  |  |  |  |  |  |  |  |  |  |
| 41 |  |  |  |  |  |  |  |  |  |  |  |
| 42 |  |  |  |  |  |  |  |  |  |  |  |
| 43 |  |  |  |  |  |  |  |  |  |  |  |
| 44 |  |  |  |  |  |  |  |  |  |  |  |
| 45 |  |  |  |  |  |  |  |  |  |  |  |
| 46 |  |  |  |  |  |  |  |  |  |  |  |
| 47 |  |  |  |  |  |  |  |  |  |  |  |
| 48 |  |  |  |  |  |  |  |  |  |  |  |
| 49 |  |  |  |  |  |  |  |  |  |  |  |
| 50 |  |  |  |  |  |  |  |  |  |  |  |
| 51 |  |  |  |  |  |  |  |  |  |  |  |
| 52 |  |  |  |  |  |  |  |  |  |  |  |
| 53 |  |  |  |  |  |  |  |  |  |  |  |
| 54 |  |  |  |  |  |  |  |  |  |  |  |
| 55 |  |  |  |  |  |  |  |  |  |  |  |
| 56 |  |  |  |  |  |  |  |  |  |  |  |
| 57 |  |  |  |  |  |  |  |  |  |  |  |
| 58 |  |  |  |  |  |  |  |  |  |  |  |
| 59 |  |  |  |  |  |  |  |  |  |  |  |
| 60 |  |  |  |  |  |  |  |  |  |  |  |
| 61 |  |  |  |  |  |  |  |  |  |  |  |
| 62 |  |  |  |  |  |  |  |  |  |  |  |
| 63 |  |  |  |  |  |  |  |  |  |  |  |
| 64 |  |  |  |  |  |  |  |  |  |  |  |
| 65 |  |  |  |  |  |  |  |  |  |  |  |
| 66 |  |  |  |  |  |  |  |  |  |  |  |
| 67 |  |  |  |  |  |  |  |  |  |  |  |
| 68 |  |  |  |  |  |  |  |  |  |  |  |
| 69 |  |  |  |  |  |  |  |  |  |  |  |
| 70 |  |  |  |  |  |  |  |  |  |  |  |
| 71 |  |  |  |  |  |  |  |  |  |  |  |
| 72 |  |  |  |  |  |  |  |  |  |  |  |
| 73 |  |  |  |  |  |  |  |  |  |  |  |
| 74 |  |  |  |  |  |  |  |  |  |  |  |
| 75 |  |  |  |  |  |  |  |  |  |  |  |
| 76 |  |  |  |  |  |  |  |  |  |  |  |
| 77 |  |  |  |  |  |  |  |  |  |  |  |
| 78 |  |  |  |  |  |  |  |  |  |  |  |
| 79 |  |  |  |  |  |  |  |  |  |  |  |
| 80 |  |  |  |  |  |  |  |  |  |  |  |
| 81 |  |  |  |  |  |  |  |  |  |  |  |
| 82 |  |  |  |  |  |  |  |  |  |  |  |
| 83 |  |  |  |  |  |  |  |  |  |  |  |
| 84 |  |  |  |  |  |  |  |  |  |  |  |
| 85 |  |  |  |  |  |  |  |  |  |  |  |
| 86 |  |  |  |  |  |  |  |  |  |  |  |
| 87 |  |  |  |  |  |  |  |  |  |  |  |
| 88 |  |  |  |  |  |  |  |  |  |  |  |
| 89 |  |  |  |  |  |  |  |  |  |  |  |
| 90 |  |  |  |  |  |  |  |  |  |  |  |
| 91 |  |  |  |  |  |  |  |  |  |  |  |
| 92 |  |  |  |  |  |  |  |  |  |  |  |
| 93 |  |  |  |  |  |  |  |  |  |  |  |
| 94 |  |  |  |  |  |  |  |  |  |  |  |
| 95 |  |  |  |  |  |  |  |  |  |  |  |
| 96 |  |  |  |  |  |  |  |  |  |  |  |
| 97 |  |  |  |  |  |  |  |  |  |  |  |
| 98 |  |  |  |  |  |  |  |  |  |  |  |
| 99 |  |  |  |  |  |  |  |  |  |  |  |
| 100 |  |  |  |  |  |  |  |  |  |  |  |
| 101 |  |  |  |  |  |  |  |  |  |  |  |
| 102 |  |  |  |  |  |  |  |  |  |  |  |
| 103 |  |  |  |  |  |  |  |  |  |  |  |
| 104 |  |  |  |  |  |  |  |  |  |  |  |
| 105 |  |  |  |  |  |  |  |  |  |  |  |
| 106 |  |  |  |  |  |  |  |  |  |  |  |
| 107 |  |  |  |  |  |  |  |  |  |  |  |
| 108 |  |  |  |  |  |  |  |  |  |  |  |
| 109 |  |  |  |  |  |  |  |  |  |  |  |
| 110 |  |  |  |  |  |  |  |  |  |  |  |
| 111 |  |  |  |  |  |  |  |  |  |  |  |
| 112 |  |  |  |  |  |  |  |  |  |  |  |
| 113 |  |  |  |  |  |  |  |  |  |  |  |
| 114 |  |  |  |  |  |  |  |  |  |  |  |
| 115 |  |  |  |  |  |  |  |  |  |  |  |
| 116 |  |  |  |  |  |  |  |  |  |  |  |
| 117 |  |  |  |  |  |  |  |  |  |  |  |
| 118 |  |  |  |  |  |  |  |  |  |  |  |
| 119 |  |  |  |  |  |  |  |  |  |  |  |
| 120 |  |  |  |  |  |  |  |  |  |  |  |
| 121 |  |  |  |  |  |  |  |  |  |  |  |
| 122 |  |  |  |  |  |  |  |  |  |  |  |
| 123 |  |  |  |  |  |  |  |  |  |  |  |
| 124 |  |  |  |  |  |  |  |  |  |  |  |
| 125 |  |  |  |  |  |  |  |  |  |  |  |
| 126 |  |  |  |  |  |  |  |  |  |  |  |
| 127 |  |  |  |  |  |  |  |  |  |  |  |
| 128 |  |  |  |  |  |  |  |  |  |  |  |
| 129 |  |  |  |  |  |  |  |  |  |  |  |
| 130 |  |  |  |  |  |  |  |  |  |  |  |
| 131 |  |  |  |  |  |  |  |  |  |  |  |
| 132 |  |  |  |  |  |  |  |  |  |  |  |
| 133 |  |  |  |  |  |  |  |  |  |  |  |
| 134 |  |  |  |  |  |  |  |  |  |  |  |
| 135 |  |  |  |  |  |  |  |  |  |  |  |
| 136 |  |  |  |  |  |  |  |  |  |  |  |
| 137 |  |  |  |  |  |  |  |  |  |  |  |
| 138 |  |  |  |  |  |  |  |  |  |  |  |
| 139 |  |  |  |  |  |  |  |  |  |  |  |
| 140 |  |  |  |  |  |  |  |  |  |  |  |
| 141 |  |  |  |  |  |  |  |  |  |  |  |
| 142 |  |  |  |  |  |  |  |  |  |  |  |
| 143 |  |  |  |  |  |  |  |  |  |  |  |
| 144 |  |  |  |  |  |  |  |  |  |  |  |
| 145 |  |  |  |  |  |  |  |  |  |  |  |
| 146 |  |  |  |  |  |  |  |  |  |  |  |
| 147 |  |  |  |  |  |  |  |  |  |  |  |
| 148 |  |  |  |  |  |  |  |  |  |  |  |
| 149 |  |  |  |  |  |  |  |  |  |  |  |
| 150 |  |  |  |  |  |  |  |  |  |  |  |
| 151 |  |  |  |  |  |  |  |  |  |  |  |
| 152 |  |  |  |  |  |  |  |  |  |  |  |
| 153 |  |  |  |  |  |  |  |  |  |  |  |
| 154 |  |  |  |  |  |  |  |  |  |  |  |
| 155 |  |  |  |  |  |  |  |  |  |  |  |
| 156 |  |  |  |  |  |  |  |  |  |  |  |
| 157 |  |  |  |  |  |  |  |  |  |  |  |
| 158 |  |  |  |  |  |  |  |  |  |  |  |
| 159 |  |  |  |  |  |  |  |  |  |  |  |
| 160 |  |  |  |  |  |  |  |  |  |  |  |
| 161 |  |  |  |  |  |  |  |  |  |  |  |
| 162 |  |  |  |  |  |  |  |  |  |  |  |
| 163 |  |  |  |  |  |  |  |  |  |  |  |
| 164 |  |  |  |  |  |  |  |  |  |  |  |
| 165 |  |  |  |  |  |  |  |  |  |  |  |
| 166 |  |  |  |  |  |  |  |  |  |  |  |
| 167 |  |  |  |  |  |  |  |  |  |  |  |
| 168 |  |  |  |  |  |  |  |  |  |  |  |
| 169 |  |  |  |  |  |  |  |  |  |  |  |
| 170 |  |  |  |  |  |  |  |  |  |  |  |
| 171 |  |  |  |  |  |  |  |  |  |  |  |
| 172 |  |  |  |  |  |  |  |  |  |  |  |
| 173 |  |  |  |  |  |  |  |  |  |  |  |
| 174 |  |  |  |  |  |  |  |  |  |  |  |
| 175 |  |  |  |  |  |  |  |  |  |  |  |
| 176 |  |  |  |  |  |  |  |  |  |  |  |
| 177 |  |  |  |  |  |  |  |  |  |  |  |
| 178 |  |  |  |  |  |  |  |  |  |  |  |
| 179 |  |  |  |  |  |  |  |  |  |  |  |
| 180 |  |  |  |  |  |  |  |  |  |  |  |
| 181 |  |  |  |  |  |  |  |  |  |  |  |
| 182 |  |  |  |  |  |  |  |  |  |  |  |
| 183 |  |  |  |  |  |  |  |  |  |  |  |
| 184 |  |  |  |  |  |  |  |  |  |  |  |
| 185 |  |  |  |  |  |  |  |  |  |  |  |
| 186 |  |  |  |  |  |  |  |  |  |  |  |
| 187 |  |  |  |  |  |  |  |  |  |  |  |
| 188 |  |  |  |  |  |  |  |  |  |  |  |
| 189 |  |  |  |  |  |  |  |  |  |  |  |
| 190 |  |  |  |  |  |  |  |  |  |  |  |
| 191 |  |  |  |  |  |  |  |  |  |  |  |
| 192 |  |  |  |  |  |  |  |  |  |  |  |
| 193 |  |  |  |  |  |  |  |  |  |  |  |
| 194 |  |  |  |  |  |  |  |  |  |  |  |
| 195 |  |  |  |  |  |  |  |  |  |  |  |
| 196 |  |  |  |  |  |  |  |  |  |  |  |
| 197 |  |  |  |  |  |  |  |  |  |  |  |
| 198 |  |  |  |  |  |  |  |  |  |  |  |
| 199 |  |  |  |  |  |  |  |  |  |  |  |
| 200 |  |  |  |  |  |  |  |  |  |  |  |
