# Supplementary material for: Method for Observing SMOKing and vaping bEhaviours (MOSMOKE): development and validation of a systematic observation tool
Source: BMJ Open. 2025 Jul 13;15(7):e105510. doi: 10.1136/bmjopen-2025-105510 (PMC12258348; doi:10.1136/bmjopen-2025-105510)
Supplement: online supplemental file 3 [file bmjopen-15-7-s003.docx]

**DATE / DAY: _____________________________________________ SITE: _______________________________________________ OBSERVER: _______________**

**WEATHER/ COMMENTS:** ___________________________________________________________________________________________________________________

*Include the duration of any precipitation e.g. ‘Rain from 10.20-10.45am. Also make a note of any other potentially important observations.*

| **AGE GROUP** | **TOTAL COUNT** | **SMOKING** | | **VAPING** | |
| --- | --- | --- | --- | --- | --- |
|  |  | **Holding cigarette** | **Inhaling cigarette** | **Holding vape** | **Inhaling from a vape** |
| **Infant** |  |  |  |  |  |
| **Child** |  |  |  |  |  |
| **Teen** |  |  |  |  |  |
| **Adult** |  |  |  |  |  |
| **Older Adult** |  |  |  |  |  |
| **Group / Busy** |  |  |  |  |  |
|  | **Frequencies by age group**  Infant: Child: Teen: Adult: Older Adult: | | | | |

| **LITTER AUDIT** | **FREQUENCY** |
| --- | --- |
| **Cigarette butts** |  |
| **Cigarette packaging / tobacco pouches** |  |
| **Rolling paper and filter waste** |  |
| **Lighters** |  |
| **Vape pens / cartridges / pods** |  |
| **Vape stickers** |  |
