# Supplementary material for: Method for Observing SMOKing and vaping bEhaviours (MOSMOKE): development and validation of a systematic observation tool
Source: BMJ Open. 2025 Jul 13;15(7):e105510. doi: 10.1136/bmjopen-2025-105510 (PMC12258348; doi:10.1136/bmjopen-2025-105510)
Supplement: online supplemental file 4 [file bmjopen-15-7-s004.docx]

**Supplementary material 4.** Completed STROBE (Strengthening the Reporting of Observational Studies in Epidemiology) checklist

This checklist was elaborated using formal items recommended for cross-sectional studies from STROBE statement (https://www.strobe-statement.org).

|  | Item No | Recommendation | Respected? | Comments and quotes |
| --- | --- | --- | --- | --- |
| **Title and abstract** | 1 | (*a*) Indicate the study’s design with a commonly used term in the title or the abstract | Yes | Study design is indicated in the methods section of the abstract:  “A 2x2 study design was used” (page 2) |
|  |  | (*b*) Provide in the abstract an informative and balanced summary of what was done and what was found | Yes | This information is stated in the study abstract (study aim described, methods and results described) (page 2) |
| Introduction | | |  |  |
| Background/rationale | 2 | Explain the scientific background and rationale for the investigation being reported | Yes | Rationale and existing literature are stated in the introduction section (pages 5-7) |
| Objectives | 3 | State specific objectives, including any prespecified hypotheses | Yes | A statement at the end of the introduction specifies the aims and specific objectives.  “This study aimed to develop the MOSMOKE tool and test its reliability and validity. The specific objectives were to: (1) develop a new systematic observation tool for assessing smoking and vaping behaviours in public spaces; (2) assess inter-rater reliability between pairs of observers; and (3) evaluate criterion-related validity by examining differences in smoking and vaping behaviours in relation to environmental characteristics hypothesised to be associated with these behaviours.” (page 7) |
| Methods | | |  |  |
| Study design | 4 | Present key elements of study design early in the paper | Yes | Study design is stated in the methods. Key elements are described in the methods.  “Observations took place at four different sites within St Peter’s Square. These sites were selected based on two key environmental factors, creating a 2x2 factorial design to assess the criterion-related validity of MOSMOKE - that is, how well the MOSMOKE tool’s measurements predict or correlate with an external criterion.” (pages 11-12) |
| Setting | 5 | Describe the setting, locations, and relevant dates, including periods of recruitment, exposure, follow-up, and data collection | Yes | Setting, contexts, relevant dates, are described in the methods section.  “The study was conducted in St Peter’s Square, a busy public square in central Manchester, UK. Manchester is an ideal setting to test this new methodology because of its above-average adult smoking prevalence rate; the latest published UK Official for National Statistics (ONS) data for 2023 suggest that 12.5% of adults aged 18 years and over in Greater Manchester currently smoke cigarettes, compared with the 11.9% national average in the UK (26). St Peter’s Square was chosen as it provided sufficient footfall to collect a large volume of data. Further, Manchester City Council have identified this square as a potential site for future outdoor smoke-free public spaces (27).” (pages 7 and 8)  “This was an observational study using the MOSMOKE tool to collect data over four weekdays (Friday, Monday, Tuesday, and Friday) in March 2024. Observations took place at four different sites within St Peter’s Square. These sites were selected based on two key environmental factors, creating a 2x2 factorial design to assess the criterion-related validity of MOSMOKE - that is, how well the MOSMOKE tool’s measurements predict or correlate with an external criterion.” (page 11) |
| Participants | 6 | (*a*) Give the eligibility criteria, and the sources and methods of selection of participants | Yes | Study population is described in the methods section (under “MOSMOKE tool”), as well as selection criteria:  “The MOSMOKE tool was used to systematically code smoking and vaping behaviours of all individuals entering a clearly defined target area during pre-specified observation periods. Before data collection began, all observers visited the site together to agree on the exact boundaries of the target area, ensuring consistency in determining which individuals fell within the designated space. This process was crucial to avoid ambiguity when coding behaviours, as only individuals and behaviours within the target area were included in the observations.  During each observation period, observers used MOSMOKE to code the following:   1. Smoking and vaping behaviours:  - Holding a cigarette - Holding a vape - Inhaling a cigarette (identified by arm-to-mouth movement) - Inhaling from a vape (identified by arm-to-mouth movement)  1. Estimated age group (based on visual cues such as appearance, clothing, and mobility):  - Infant (baby or toddler in a pram, sling, or other carrier) - Child (appears to be up to 12 years old) - Teen (appears to be 13-19 years old) - Adult (appears to be 20-74 years old) - Older Adult (appears to be 75+ years old)   Large groups (more than 10 individuals) were excluded from observations to minimise the risk of missing smoking or vaping behaviours, as it was difficult to accurately observe and code behaviours within such groups while simultaneously monitoring other individuals passing through the target area.” (pages 9 and 10) |
| Variables | 7 | Clearly define all outcomes, exposures, predictors, potential confounders, and effect modifiers. Give diagnostic criteria, if applicable | Yes | Standardised variable definitions were used based on the MOSMOKE tool, which are presented in methods section. As these studies focused on testing psychometrics of the tool itself, there were no potential confounders.  “1. Smoking and vaping behaviours:   - Holding a cigarette - Holding a vape - Inhaling a cigarette (identified by arm-to-mouth movement) - Inhaling from a vape (identified by arm-to-mouth movement)  1. Estimated age group (based on visual cues such as appearance, clothing, and mobility):  - Infant (baby or toddler in a pram, sling, or other carrier) - Child (appears to be up to 12 years old) - Teen (appears to be 13-19 years old) - Adult (appears to be 20-74 years old) - Older Adult (appears to be 75+ years old)” (page 9)   “In addition to behavioural observations, MOSMOKE included an environmental audit of tobacco and vape-related litter. This audit recorded the presence of the following items: cigarette butts, packaging, rolling paper/filter waste, lighters, vape pens, cartridges, pods, and stickers.” (page 10) |
| Data sources/ measurement | 8* | For each variable of interest, give sources of data and details of methods of assessment (measurement). Describe comparability of assessment methods if there is more than one group | Yes | Data collection and measurement was the same for all variables of the MOSMOKE tool and is described in the methods section. Data assessment methods are described in detail under the “MOSMOKE tool” subsection in the methods.  “The MOSMOKE tool was used to systematically code smoking and vaping behaviours of all individuals entering a clearly defined target area during pre-specified observation periods. Before data collection began, all observers visited the site together to agree on the exact boundaries of the target area, ensuring consistency in determining which individuals fell within the designated space. This process was crucial to avoid ambiguity when coding behaviours, as only individuals and behaviours within the target area were included in the observations.  During each observation period, observers used MOSMOKE to code the following:   1. Smoking and vaping behaviours:  - Holding a cigarette - Holding a vape - Inhaling a cigarette (identified by arm-to-mouth movement) - Inhaling from a vape (identified by arm-to-mouth movement)  1. Estimated age group (based on visual cues such as appearance, clothing, and mobility):  - Infant (baby or toddler in a pram, sling, or other carrier) - Child (appears to be up to 12 years old) - Teen (appears to be 13-19 years old) - Adult (appears to be 20-74 years old) - Older Adult (appears to be 75+ years old)   Large groups (more than 10 individuals) were excluded from observations to minimise the risk of missing smoking or vaping behaviours, as it was difficult to accurately observe and code behaviours within such groups while simultaneously monitoring other individuals passing through the target area  In addition to behavioural observations, MOSMOKE included an environmental audit of tobacco and vape-related litter. This audit recorded the presence of the following items: cigarette butts, packaging, rolling paper/filter waste, lighters, vape pens, cartridges, pods, and stickers.” (pages 9 and 10) |
| Bias | 9 | Describe any efforts to address potential sources of bias | Yes | In the “Strengths and limitations” sub-section in the discussion, we reflect on potential limitations in the methods used:  “Since participant surveys were not conducted, there is a risk that certain behaviours may have been misclassified. For example, heated tobacco products - which use real tobacco but resemble vaping devices - might have been coded as vaping, even though they are more similar to cigarettes due to their use of tobacco. Observers reported rare instances where it was difficult to distinguish vaping devices from other handheld items (e.g., mobile phones), but this did not appear to substantially affect inter-rater reliability, which remained ‘good’ for identifying individuals holding a vape across all observer pairs (see Table 1). The study was also limited to daylight hours for ethical reasons, so it is unclear if smoking and vaping behaviours would be observed as reliably at night.  Another limitation relates to the coding of estimated age groups, which showed lower inter-rater reliability for the ‘Teen’ category. Although the same procedure for coding age group has shown high reliability in previous studies using the MOHAWk tool, reliability was lower here. This may be partly due to fewer contextual cues, as data collection for inter-rater reliability took place during the UK half term when many teens were not wearing school or college uniforms, which can help distinguish them from adults. Additionally, observer training focused more on smoking and vaping behaviours, which might have reduced attention to estimating age. Future studies aiming to assess differences by age group should place greater emphasis on practice and training for estimating age to improve reliability.” (pages 21 and 22) |
| Study size | 10 | Explain how the study size was arrived at | Partially | The methods section describes justification for the observation scheduling:  “Previous research indicates that shortened observation schedules (e.g., two days with four observation periods per day) can provide reliable estimates of activity in public spaces (26).” (page 12) |
| Quantitative variables | 11 | Explain how quantitative variables were handled in the analyses. If applicable, describe which groupings were chosen and why | Yes | We explained which variables were compared for the analyses:  “Criterion-related validity was assessed using Mann-Whitney U tests to compare smoking and vaping behaviours across sites, based on the presence of a smoking bin (bin vs. no bin) and adjacency to an office (office vs. no office).” (page 13) |
| Statistical methods | 12 | (*a*) Describe all statistical methods, including those used to control for confounding | Yes | These are described in the methods section.  “Inter-rater reliability between each pair of observers was assessed using two-way mixed, single measure, consistency intraclass correlation coefficients (ICCs). ICCs can be interpreted as <0.5=“poor”; 0.5–0.75=“moderate”; 0.76–0.9=“good”; and >0.9=“excellent” (35).” (page 12) |
|  |  | (*b*) Describe any methods used to examine subgroups and interactions | N/A | N/A |
|  |  | (*c*) Explain how missing data were addressed | N/A | N/A |
|  |  | (*d*) If applicable, describe analytical methods taking account of sampling strategy | N/A | N/A |
|  |  | (*e*) Describe any sensitivity analyses | N/A | N/A |
| Results | | |  |  |
| Participants | 13* | (a) Report numbers of individuals at each stage of study—eg numbers potentially eligible, examined for eligibility, confirmed eligible, included in the study, completing follow-up, and analysed | Yes | This is described at the beginning of the results section:  “Across the four sites, a total of 14,980 individuals were observed during 32 hours of data collection.” (pages 13 and 14) |
|  |  | (b) Give reasons for non-participation at each stage | N/A | This is not applicable to unobtrusive systematic observation. |
|  |  | (c) Consider use of a flow diagram | N/A | Use of a flow diagram was not deemed appropriate |
| Descriptive data | 14* | (a) Give characteristics of study participants (eg demographic, clinical, social) and information on exposures and potential confounders | Yes | Table 2 describes the observed characteristics of participants for the observations (page 16). |
|  |  | (b) Indicate number of participants with missing data for each variable of interest | N/A | N/A |
| Outcome data | 15* | Report numbers of outcome events or summary measures | Yes | All numbers for the observations are reported in Tables 1-3 (pages 15-18). |
| Main results | 16 | (*a*) Give unadjusted estimates and, if applicable, confounder-adjusted estimates and their precision (eg, 95% confidence interval). Make clear which confounders were adjusted for and why they were included | Yes | All unadjusted estimates, and 95% confidence intervals, are reported in Table 1 (page 15) and as follows:  “There were significantly more smoking behaviours observed at sites with a smoking bin (n=204) compared to sites without a bin (n=141) (*U*=1564.0, *p*=.02, *r*=.21). Similarly, more smoking behaviours were observed at office sites (n=212) compared to non-office sites (n=133) (*U*=1478.00, *p*=.006, *r*=.24).  In contrast, vaping behaviours did not differ significantly across these site characteristics. There were no significant differences between sites with a smoking bin (n=254) and those without (n=316) (*U*=1678.50, *p*=.08, *r*=.16), or between office (n=318) and non-office sites (n=252) (*U*=1664.0, *p*=.07, *r*=.25).” (page 14)  There were no adjustment estimates or confounders. |
|  |  | (*b*) Report category boundaries when continuous variables were categorized | N/A | N/A |
|  |  | (*c*) If relevant, consider translating estimates of relative risk into absolute risk for a meaningful time period | N/A | N/A |
| Other analyses | 17 | Report other analyses done—eg analyses of subgroups and interactions, and sensitivity analyses | N/A | N/A |
| Discussion | | |  |  |
| Key results | 18 | Summarise key results with reference to study objectives | Yes | Key results are described at the beginning of Discussion section. They are also summarised in the conclusion.  “This study provides preliminary evidence that MOSMOKE is a reliable and valid tool for assessing smoking and vaping behaviours in public spaces. The tool showed high inter-rater reliability between observers, indicating that MOSMOKE is intuitive and requires minimal training for effective application. Observed smoking and vaping at different sites aligned with the pre-specified hypotheses, providing preliminary evidence that the codes used in MOSMOKE are valid.” (page 19)  “This study provides preliminary evidence that MOSMOKE is a reliable and valid tool for systematically observing smoking and vaping behaviours in public spaces. The tool is freely available for use and includes a detailed instruction manual, observation form, data summary form, all provided in Supplementary Materials 1-3. MOSMOKE is particularly valuable for unobtrusively assessing how smoking and vaping behaviours vary across different environmental contexts and for evaluating changes in response to policies and interventions.” (page 24) |
| Limitations | 19 | Discuss limitations of the study, taking into account sources of potential bias or imprecision. Discuss both direction and magnitude of any potential bias | Yes | Description of limitations is done under “Strengths and limitations” heading in the discussion.  “However, there are some limitations. The relatively small sample size resulted in wide confidence intervals for some reliability estimates (Table 1). Since participant surveys were not conducted, there is a risk that certain behaviours may have been misclassified. For example, heated tobacco products - which use real tobacco but resemble vaping devices - might have been coded as vaping, even though they are more similar to cigarettes due to their use of tobacco. Observers reported rare instances where it was difficult to distinguish vaping devices from other handheld items (e.g., mobile phones), but this did not appear to substantially affect inter-rater reliability, which remained ‘good’ for identifying individuals holding a vape across all observer pairs (see Table 1). The study was also limited to daylight hours for ethical reasons, so it is unclear if smoking and vaping behaviours would be observed as reliably at night.  Another limitation relates to the coding of estimated age groups, which showed lower inter-rater reliability for the ‘Teen’ category. Although the same procedure for coding age group has shown high reliability in previous studies using the MOHAWk tool, reliability was lower here. This may be partly due to fewer contextual cues, as data collection for inter-rater reliability took place during the UK half term when many teens were not wearing school or college uniforms, which can help distinguish them from adults. Additionally, observer training focused more on smoking and vaping behaviours, which might have reduced attention to estimating age. Future studies aiming to assess differences by age group should place greater emphasis on practice and training for estimating age to improve reliability.  Despite these limitations, this initial work has developed a reliable observation measure that provides an important foundation for future research to determine whether these potential limitations pertain.” (pages 21 and 22) |
| Interpretation | 20 | Give a cautious overall interpretation of results considering objectives, limitations, multiplicity of analyses, results from similar studies, and other relevant evidence | Yes | References were added where possible and discussed under “Comparison with prior work” (pages 19 and 20). Limitations were considered in the discussion under “Strengths and limitations” (pages 21 and 22). |
| Generalisability | 21 | Discuss the generalisability (external validity) of the study results | Yes | Issues of generalisability were discussed under “Strengths and limitations” (pages 21 and 22) and “Future research” (page 23) headings in the discussion. |
| Other information | | |  |  |
| Funding | 22 | Give the source of funding and the role of the funders for the present study and, if applicable, for the original study on which the present article is based | Yes | Funding information were described under “Declarations”.  “JB is funded by a Leverhulme Trust Early Career Fellowship (ECF-2024-603). The views expressed in this paper are those of the authors and not necessarily those of the Leverhulme Trust.” (page 23) |

*Give information separately for exposed and unexposed groups.
