# Supplementary material for: Method for Observing SMOKing and vaping bEhaviours (MOSMOKE): development and validation of a systematic observation tool
Source: BMJ Open. 2025 Jul 13;15(7):e105510. doi: 10.1136/bmjopen-2025-105510 (PMC12258348; doi:10.1136/bmjopen-2025-105510)
Supplement: online supplemental file 5 [file bmjopen-15-7-s005.docx]

**Supplementary material 5.** Observation schedule for evaluating criterion-related validity and examining age-related differences

| **Date and time** | | **Office sites** | | **No office sites** | |
| --- | --- | --- | --- | --- | --- |
|  |  | **Site 1 (bin)** | **Site 2 (no bin)** | **Site 3 (bin)** | **Site 4 (no bin)** |
| Friday 1st March 2024 | 10.30am-11.30am | Observer SB |  |  | Observer JB |
|  | 12pm-1pm | Observer SB |  |  | Observer JB |
|  | 2pm-3pm |  | Observer JB | Observer SB |  |
|  | 3.30pm-4.30pm |  | Observer JB | Observer SB |  |
| Monday 4th March 2024 | 10.30am-11.30am |  | Observer SB | Observer AK |  |
|  | 12pm-1pm |  | Observer SB | Observer AK |  |
|  | 2pm-3pm |  | Observer AK | Observer SB |  |
|  | 3.30pm-4.30pm |  | Observer AK | Observer SB |  |
| Tuesday 5th March 2024 | 10.30am-11.30am | Observer SB |  |  | Observer AK |
|  | 12pm-1pm | Observer SB |  |  | Observer AK |
|  | 2pm-3pm |  | Observer AK | Observer SB |  |
|  | 3.30pm-4.30pm |  | Observer AK | Observer SB |  |
| Friday 8th March 2024 | 10.30am-11.30am |  | Observer SB | Observer JB |  |
|  | 12pm-1pm |  | Observer SB | Observer JB |  |
|  | 2pm-3pm | Observer AK |  |  | Observer SB |
|  | 3.30pm-4.30pm | Observer AK |  |  | Observer SB |
