# Supplementary material for: Method for Observing SMOKing and vaping bEhaviours (MOSMOKE): development and validation of a systematic observation tool
Source: BMJ Open. 2025 Jul 13;15(7):e105510. doi: 10.1136/bmjopen-2025-105510 (PMC12258348; doi:10.1136/bmjopen-2025-105510)
Supplement: Supplementary file 6 [file bmjopen-2025-105510.reviewer_comments.pdf]

PEER REVIEW HISTORY

BMJ Open publishes all reviews undertaken for accepted manuscripts. Reviewers are asked to complete a checklist review form and are provided with free text boxes to elaborate on their assessment. These free text comments are reproduced below.

This paper was submitted to another journal from BMJ but declined for publication following peer review. The authors addressed the reviewers' comments and submitted the revised paper to BMJ Open. The paper was subsequently accepted for publication at BMJ Open.

ARTICLE DETAILS

Title (Provisional)

Method for Observing SMOKing and vaping bEhaviours (MOSMOKE): development and validation of a systematic observation tool

Authors

Benton, Jack S; Berg, Sofia; Kapadi, Arbaz; Bendel, Neil; Jerram, Julie; French, David

---

VERSION 1 - REVIEW

**Reviewer**	**1**
**Name**	**Reviewer 1**
**Affiliation**	
**Date**	**28-Apr-2025**
**COI**	**No competing interest**

Comments not published pending reviewer's consent

**Reviewer**	**2**
**Name**	**Reviewer 2**
**Affiliation**	
**Date**	**28-Apr-2025**
**COI**	**No competing interest**

Comments not published pending reviewer's consent

**Reviewer**	**3**
**Name**	**Reviewer 3**

Affiliation

Date 28-Apr-2025

COI No competing interest

Comments not published pending reviewer’s consent
